# Jurymen Seldom Rule Against a Person That They Like: The Relationship Between Emotions Towards a Defendant, the Understanding of Case Facts, and Juror Judgments in Civil Trials

**DOI:** 10.3390/bs15070965

**Published:** 2025-07-16

**Authors:** Hannah J. Phalen, Taylor C. Bettis, Samantha R. Bean, Jessica M. Salerno

**Affiliations:** 1 Department of Psychology, University of Wyoming, 1000 E University Ave, Laramie, WY 82071, USA; 2 Department of Psychology, University of Kentucky, 503 Library Dr, Lexington, KY 40508, USA; t.bettis@uky.edu; 3 School of Interdisciplinary Forensics, Arizona State University, 4701 W Thunderbird Rd, Glendale, AZ 85306, USA; 4 Department of Psychology, Cornell University, 616 Thurston Ave., Ithaca, NY 14853, USA

**Keywords:** emotion, juror decision-making, civil jury, coherence-based reasoning, social cognition

## Abstract

Legal actors often discuss emotion-based decisions and reasoned evaluation of the facts as distinct and opposite methods through which jurors can reach conclusions. However, research suggests that emotion can have an indirect effect on juror decisions by changing the way that jurors evaluate the facts of the case. In three studies (*N* = 713, *N* = 677, *N* = 651), we tested whether mock jurors’ negative moral emotions towards the defendant predicted their evaluations of *unrelated* case evidence and in turn their case judgments and whether judicial rehabilitation could reduce this effect. Participants read a civil case and were randomly assigned to either receive judicial rehabilitation or not. Then, they completed measures relating to their negative moral emotions towards the defendant, their agreement with plaintiff and defense evidence, and case judgments. When participants reported increased negative emotions towards the defendant, they agreed more with unrelated plaintiff evidence and less with unrelated defense evidence. In turn, they voted liable more often and awarded more in damages. Judicial rehabilitation did not reduce this effect. This research provides support for the idea that there is a more complicated relationship between emotion and decisions than legal actors suggest. Specifically, negative emotions towards the defendant are associated with a pro-plaintiff evaluation of evidence and pro-plaintiff judgments.

## 1. Introduction

After convicting Elizabeth Holmes on four counts, acquitting her on four counts, and failing to reach a verdict on the additional three counts, one juror expressed that he struggled to convict because “it’s tough to convict somebody, especially somebody so likable” (Cohen, 2022). In contrast, the foreman in the case against Ahmaud Arbery’s killers described the defendants as full of hatred and unremorseful (Associated Press, 2022). Attorneys have long recognized the importance of a likeable defendant. Even in 1935, famous American trial attorney, Clarence Darrow argued that “[j]urymen seldom convict a person that they like, or acquit one that they dislike…facts regarding the crime are relatively unimportant” (Wistrich et al., 2015). In this paper, we present an empirical test of Darrow’s assertion by testing whether mock jurors assign importance to facts in line with their feelings about the defendant. Specifically, in an analysis of secondary measures from three studies (Salerno et al., 2021), we examine whether mock jurors’ emotional reactions to a defendant predict the way that they interpret the facts and, in turn, their verdicts.

### 1.1. Psychological Research on the Relationship Between Emotions and Decisions

Traditional dual-process models (e.g., J. D. Greene, 2023; Kahneman & Frederick, 2002; Wason & Evans, 1974) often categorize judgments as either emotional or reasoned. Specifically, early research suggests that people either engage in intuitive (system 1) processes or reasoning (system 2) processes. Intuitive processes are fast and automatic and generally involve emotional responses. In contrast, reasoning processes tend to be slower and more reflective. Similarly, morality researchers propose that moral judgments stem from a similar dual-process system where deontological judgments rely on emotional responses where utilitarian decisions rely on cognition and reason (J. Greene, 2005; J. Greene & Haidt, 2002; for review, see Dahò, 2025). This characterization of judgments as either emotional or reasoned stems from a long-held belief that emotion and cognition are distinct and opposite (Damasio, 1994; Levine, 2022). However, recent neuroscience research suggests that emotion and cognition are not opposites; instead, the two work as partners to develop organized and coherent decisions (for review, see Levine, 2022).

Further, a strict characterization of judgments as either emotional or reasoned ignores the ways in which emotion can still affect decisions when people are working hard to make reasoned judgments. Indeed, researchers suggest that system 1 and system 2 processes can operate concurrently and compete for control over responses (Kahneman & Frederick, 2002). Further, fuzzy-trace theory proposes that people first form gist representations (which might be influenced by emotion) that anchor later judgments (Reyna, 1992; Rivers et al., 2008).

Today, psychologists largely recognize that emotions are an important and essential element of decision-making (Lerner et al., 2015; Lerner et al., 2023; van Kleef & Côté, 2022). Indeed, research suggests that emotions play an important role in decision-making across a variety of domains from the law (Salerno, 2021), to healthcare (Ferrer & Mendes, 2018), to education (Wang, 2021), and beyond. Today, theories of emotion and decision-making are often broken into two categories: valence-based theories and specific-emotion theories.

Valence-based theories focus on the differential impact of positive and negative valanced emotions on decisions. Valence-based theories focus on direct and indirect impacts of emotions on decisions. Emotions might directly impact judgments if people making a judgment consult how they are feeling, and use their feeling as information (i.e., the affect-as-information heuristic, Schwarz & Clore, 1983; Schwarz, 2012). Further, people’s emotional reactions to information can indirectly impact decisions by influencing what information they pay attention to, recall, and how they interpret and evaluate that subsequent information (e.g., Affect Infusion Model, Forgas, 1995; Culpable Control Model, Alicke, 2000). For example, when people are exposed to an act that elicits negative emotion (e.g., a defendant being accused of harming another person), they can engage in “blame validation processes”, which include paying selective attention to other information that is consistent with those negative feelings to justify blaming someone for the harm (Alicke, 2000).

In contrast, specific-emotion theories focus on the unique impacts of different emotions. Researchers propose that distinct moral emotions might have different impacts on decision-making (Haidt, 2003; Lerner et al., 2015). The appraisal-tendency framework (Lerner & Keltner, 2000) proposes that an emotion’s intensity and qualitative character changes its motivational properties and, in turn, the response to that emotion. For example, feeling some emotions, such as anger and disgust, might prime people to blame someone, which can lead to people interpreting evidence in line with that need to blame. In contrast, other emotions, such as sadness and sympathy, might prime people to help, and can lead to people interpreting evidence in line with that need to help. Some moral emotions, however, such as guilt, are not characterized by specific motivational properties (Gangemi et al., 2025).

More recently, researchers have proposed a model that integrates valence-based theories and specific-emotion theories: the emotion-imbued choice model (Lerner et al., 2015, 2023). This model posits that current specific emotions can influence decisions both directly and indirectly by altering the decision-maker’s evaluation of information, processing, and their implicit goals.

The emotion-imbued choice model is unique in that the relationship between emotions and decisions is bidirectional. That is, the valence-based and specific-emotion theories offer a unidirectional explanation for the relationship between emotions and decisions: Emotions inform how someone processes and interprets other information, which then determines their decision. However, the emotion-imbued choice model suggests that in addition to emotions informing evaluations of information, that information can also influence emotions. In line with this model, researchers have suggested that people evaluate their emotions and their understanding of information simultaneously to reach a conclusion that maximizes Cognitive Consistency (Simon et al., 2015, 2020; for review see Simon & Read, 2025).

### 1.2. The Role of Cognitive Consistency in Decision-Making

The idea of Cognitive Consistency is seen in many seminal psychological theories (e.g., Balance Theory, Heider, 1946; Cognitive Dissonance Theory, Festinger, 1957; Affective-Cognitive Consistency Theory, Rosenberg, 1960) that all propose that people will revise their beliefs to be consistent with their feelings and actions. While most early research on Cognitive Consistency was unidirectional, more recent research into cognitive processes proposes that the principles of Cognitive Consistency can operate in a bidirectional nature (e.g., The Coherence Model of Cognitive Consistency; Spellman, 1996). More specifically, both computer simulations (Shultz & Lepper, 1996; Spellman, 1996) and laboratory-based research (Glöckner & Witteman, 2010; Russo et al., 2008) suggest that people revise the strength and direction of previously held beliefs when new information is introduced, in order to maximize the coherence between the prior beliefs and the new information.

However, most of this research examines the effect of coherence in “cold cognitions”, or the interpretations of facts, probabilities, etc. For example, confirmation bias is a classic example of seeking coherence in a cold cognition (Simon & Read, 2025). Broadly, confirmation bias is the tendency to seek out and interpret information in a manner that confirms and reinforces prior beliefs (Oswald & Grosjean, 2004; see also Wason, 1960). In a classic study on confirmation bias, Lord et al. (1979) found that participants reported more extreme positions after (compared to before) exposure to mixed evidence on the effectiveness of the death penalty. Further, participants were more likely to favorably evaluate evidence that confirmed their prior beliefs and negatively evaluate evidence that contradicted their prior beliefs. Thus, participants demonstrated strong coherence in that their beliefs and judgments of new evidence coalesced, and they revised their beliefs in line with their judgments of new evidence.

But the coherence effect can also be used to explain how emotions and other “hot cognitions” can influence decisions. In one study, for example, when participants felt angrier towards a person in a relationship, they evaluated the person as less committed to the relationship and they evaluated the person’s actions as more dishonest (Simon et al., 2015; Study 1). In another study, when participants learned information designed to increase sympathy toward a defendant (e.g., that her brother was killed by a drunk driver), they reported concordant hot and cold cognitions and were more likely to find her innocent (Simon et al., 2020). In sum, research suggests that people engage in a coherence-based reasoning process wherein they interpret facts in line with their “hot cognitions” (Simon et al., 2015, 2020; for review see Simon & Read, 2025).

One manner through which Cognitive Consistency might be achieved is through motivated reasoning, which Simon and Read (2025) argue is the strongest contributor to biased reasoning of all the hot cognitions. Broadly, motivated reasoning is the tendency to interpret new information in line with prior information or beliefs (Kunda, 1990). Motivated reasoning can manifest both as unwarranted positive evaluations of consistent information and unwarranted negative evaluations of inconsistent information. In a legal context, jurors might unfairly weigh evidence that is consistent with their emotions and prior beliefs and unfairly disregard evidence that is not consistent with their emotions and prior beliefs.

This paper will expand on previous research about the coherence effect by examining the relationship between emotions towards a defendant, mock jurors’ evaluation of case facts, and their ultimate judgments.

### 1.3. Emotion and Legal Decision-Making

There is a distinct difference in the ways that the U.S. jury system and psycho-legal scholars understand emotions and their effects on juror decision making. The U.S. jury system’s conceptualization of the effect of emotions on decision-making is largely influenced by the traditional dual-process models discussed above (Bandes, 1999; Salerno, 2021). Specifically, the U.S. jury system often considers only the direct impact of emotion on verdicts—worrying that jurors will rely only and directly on their emotional response without paying attention to evidence and reasoned arguments—characterizing emotion as the enemy of reason (Bandes, 1999; Salerno, 2021). Indeed, legal actors often treat emotion and reason as fully distinct—as evidenced by jury instructions mandating that jurors should apply the law to the facts without sympathy or passion (e.g., U.S. Eleventh Circuit District Judicial Council, 2024; U.S. Ninth Circuit Committee on Model Criminal Jury Instructions, 2022).

Psycholegal scholars take a more nuanced view of the impact of emotions in decision-making, in line with the psychological theories discussed above. Specifically, despite the focus in the legal system on the direct impact of emotion on judgments, research has also demonstrated that emotions impact legal judgments indirectly and provides support for a coherence-based model of the impact of emotion on decisions (for reviews, see Feigenson & Park, 2006; Salerno, 2021; Peter-Hagene et al., 2023).

In the criminal realm, several studies have examined the indirect impact of emotions on juror decisions. Increased anger predicts an increase in the likelihood that mock jurors sentence defendants to death (Nuñez et al., 2015). Mock jurors who experience negative emotions (compared to those who do not) judge actions as more intentional (Ask & Pina, 2011); rely more on stereotypes when deciding a case (Bodenhausen et al., 1994); and are less sensitive to strong (versus weak) defense evidence (Salerno, 2017). Other research has shown that irrelevant negative emotions towards a defendant predicted a pro-prosecution evaluation of unrelated key evidence in the case (Simon et al., 2015; Study 2). Similarly, research suggests that emotion might influence the types of questions asked in child sexual abuse interviews (Gewehr et al., 2025). One study found that civil mock jurors act in the same way. Specifically, as civil mock jurors felt increased disgust about the plaintiff’s injury, they reported increased agreement with the plaintiff’s evidence, but decreased agreement with the defense’s evidence, which in turn predicts an increased likelihood to return a verdict for the plaintiff (Salerno & Phalen, 2019).

Salerno (2021) proposes that this research is consistent with theories of coherence-based reasoning. Specifically, some case facts (e.g., photographs and descriptions of a plaintiff’s injuries) might foster negative emotions towards a defendant, which might, in turn, lead jurors to interpret other facts through an anti-defendant lens. Then, when jurors believe there is increased “evidence” against the defendant, their skewed evaluation of the facts might reinforce and strengthen their negative emotions towards the defendant. In this way, jurors will maximize coherence between their emotions and their understanding of the facts. While it might be reasonable for negative emotions towards the defendant to influence jurors’ evaluations of evidence that relates to the defendant, negative emotions towards the defendant should not influence how jurors evaluate evidence that is unrelated to the defendant. That is, if jurors believe that a defendant is immoral, they might be more willing to accept evidence that a defendant acted immorally but jurors’ beliefs about the defendant’s morality should not influence their evaluation of evidence about the plaintiff’s actions.

Therefore, this research expands on prior research on the relationship between emotions towards the defendant and evaluation of the facts by examining the association between negative moral emotions towards the defendant, their evaluations of plaintiff and defense evidence that is *unrelated* to the defendant, and case judgments (i.e., verdicts and damage awards). That is, we will test whether having negative emotional reactions to the defendant is related to evaluating other evidence in the case (unrelated to the defendant) in line with those negative emotions, to justify pro-plaintiff case judgments. Additionally, through a manipulation that was included in the original datasets, we will be investigating whether the hypothesized relationship between mock jurors’ emotional reactions to the defendant and their evaluation of other case evidence is attenuated by judicial rehabilitation.

### 1.4. Does Judicial Rehabilitation Diminish the Relationship Between Emotion and Case Judgments?

If jurors’ emotional reactions to the defendant skew the way they evaluate the other evidence and decide the case, what can courts do to intervene? One potential solution is the use of judicial rehabilitation. Judicial rehabilitation is a legal term describing a process in which a judge explicitly asks a juror if they are able and willing to set aside any potential biases to make a fair judgment. The aim of judicial rehabilitation is to encourage fair decision making by jurors, and assist in diminishing any influence that a juror’s bias may hold (*Thomas ex rel. Thomas v. Mercy Hospitals East Communities*, 2017). In this legal context, bias means any belief that a juror holds that might influence their decision.

The legal system puts great stock in judicial rehabilitation as a way to motivate jurors to be impartial and avoid being biased (Hannaford-Agor & Waters, 2004). It is possible that judicial rehabilitation might reduce juror bias by raising both awareness of that bias and concern about preventing bias—two necessary conditions for a person to mitigate their biases (Devine et al., 2012; Gawronski et al., 2006; Paluck et al., 2021). Unfortunately, most research on judicial rehabilitation in criminal cases has found it to be ineffective in diminishing the link between jurors’ pre-trial attitudes and their case judgments (Crocker & Kovera, 2010; Salerno et al., 2021). At best, research shows that debiasing instructions tend to reduce, rather than eliminate bias (Axt et al., 2018) and those effects are short-lived (Lai et al., 2016). At worst, research has found that instructing jurors to avoid being biased can backfire, resulting in jurors who act on their biases while reporting that they are impartial (Lieberman & Arndt, 2000; Salerno et al., 2021). In this paper, we investigate the potential impact of judicial rehabilitation in a different context: as a potential intervention in a civil case between negative emotions towards the defendant and jurors’ evaluation of the facts. On the one hand, judicial rehabilitation might reduce the impact of emotions on biased review of the evidence by focusing jurors on trying to avoid biases and reviewing the evidence in an impartial manner. On the other hand, judicial rehabilitation might backfire, increasing the biasing impact of emotions while falsely convincing jurors that they are unbiased.

### 1.5. Study Overview and Hypotheses

We conducted three simultaneous mock juror experiments with the same procedure and design, which we collapsed across for analyses (*N* = 2041). The experiments differed only in case scenario. In all three studies, participants read an extensive trial stimulus that included a summary of the case background, opening statements, examinations of witnesses, and closing arguments. In all studies, participants read jury instructions detailing the burden of proof and case-relevant law. The full design and procedures are discussed in the original manuscript (Salerno et al., 2021). Relevant to this article, participants were randomly assigned to either receive judicial rehabilitation or not, which included watching a video recording of a judge who asked participants whether they could put aside any biases that they have and apply the law. Participants who were in the judicial rehabilitation condition were excluded if they answered “no” (*n* = 11). After the judicial rehabilitation manipulation, participants read the case information. Then participants gave verdicts and damage awards and completed measures of their negative emotions about the defendant and their agreement with plaintiff and defense evidence that was unrelated to the defendants’ behavior.

Across all three studies, we had three hypotheses. First, we hypothesized that feeling increased negative emotions towards the defendant would be associated with an increase in the likelihood that a participant would vote liable (Hypothesis 1a) and award higher damages (Hypothesis 1b). Second, we hypothesized an indirect effect of emotions on case judgments, such that agreement with the plaintiff and defense evidence that was unrelated to the defendants’ behavior would mediate the relationship between negative emotions and case judgments. Specifically, we predicted that increased negative emotions towards the defendant would be associated with (a) increased agreement with unrelated plaintiff evidence and (b) decreased agreement with unrelated defense evidence. In turn, both increased agreement with plaintiff evidence and decreased agreement with defense evidence would be associated with an increased likelihood to vote liable (Hypothesis 2a) and award higher damages (Hypothesis 2b). Third, we hypothesized that judicial rehabilitation would not diminish this hypothesized indirect effect given its prior failures to reduce the link between jurors’ pre-existing attitudes and case judgments (Hypotheses 3a-verdicts and 3b-damage awards; Crocker & Kovera, 2010; Salerno et al., 2021).

## 2. Method

### 2.1. Participants and Procedure

As reported in the original manuscript, we recruited 2567 adults from Amazon’s Mechanical Turk (M-Turk). Research indicates that M-Turk samples are demographically diverse (compared to traditional convenience samples such as college students), yield results similar to other nationally representative samples (Coppock, 2019), and are a source of quality data (Buhrmester et al., 2018; Irvine et al., 2018). We used Cloud Research to manage the M-Turk workers, which allowed us to measure for quality assurance by screening out: (1) participants who were not in the U.S. (including participants in other countries using U.S. based VPNs); (2) participants who have been identified as using message boards to skirt attention checks; and (3) M-Turk workers with a worker score of less than 80%. Participants completed seven attention checks consisting of three checks where they were asked to select a specific answer (e.g., Strongly Agree) and four checks where they were asked to confirm their demographic information. Participants also answered a manipulation check asking them to report whether or not they saw a video from the judge, as only participants in the judicial rehabilitation condition saw a video from the judge. Additionally, immediately after reading the trial stimulus, participants answered three comprehension questions about the trial stimulus. We excluded data from 526 participants (20%; an exclusion rate typical for M-Turk samples; Goodman et al., 2013) for failing attention checks, *n* = 359, 13.99%; failing the judicial rehabilitation manipulation check, *n* = 68, 2.6%; incorrectly answering any two of three comprehension questions, *n* = 181, 7.05%; or for reporting that they could not be impartial after seeing judicial rehabilitation, *n* = 11, 0.44. Thus, the final sample size was 2041. Table 1 includes the demographic information. Participants were paid USD 4.50 and the study took approximately 45 min to complete.

**Figure 1:**
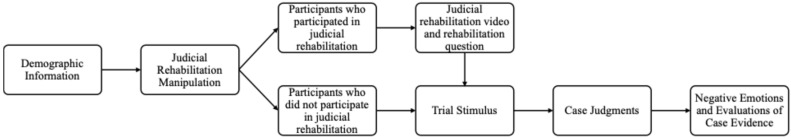
Procedure.

Figure 1 describes the procedure. In all studies, participants first completed demographic questions. Then they were randomly assigned to either participate in judicial rehabilitation or not. Participants who did not participate in judicial rehabilitation read the trial stimulus immediately after answering basic demographic questions. Participants who did participate in judicial rehabilitation viewed a brief video and answered a question (described below) before moving on to read the trial stimulus. After reading an extensive civil trial stimulus, participants made case judgments. After completing their case judgments, they self-reported the extent to which they felt negative emotions towards the defendant and the extent to which they agreed with evidence that was presented by the plaintiff and defendant. The current research was approved by the [masked university] IRB (#00001858).

### 2.2. Materials and Measures

#### 2.2.1. Judicial Rehabilitation Manipulation

Before reading the trial stimuli, participants were randomly assigned to either see judicial rehabilitation or not. When participants saw judicial rehabilitation, they saw a brief video in which a judge asked whether the participant could put aside their biases and apply the law. This video was created using the real question that judges will ask jurors during jury selection. Participants responded with either *yes* or *no*. Although we intended to analyze how participants who reported that they could not be impartial differed from participants who reported that they could be impartial, only 11 participants responded that they could not be impartial. Thus, we excluded these participants from the analyses. However, this low number of participants who identified their own biases speaks to the potential flaws with judicial rehabilitation.

#### 2.2.2. Trial Stimulus

In all three studies, the trial stimuli were based on actual civil cases argued in American courts and were quite extensive. The trial stimuli are available online at https://osf.io/685qa/ (accessed on 15 July 2025). While our goal was to ensure that our findings generalized across diverse cases of different contexts and evidence strength, we did not expect the hypothesized pattern of findings to differ across cases. Therefore, to reduce redundancy and increase statistical power, we collapsed response data across cases. We report the results of analyses separately for each case in Supplemental Materials (pp. 5–14, Tables S6–S9, Figures S2–S7), which largely replicate what is reported below.

We intentionally selected trial stimuli that varied in their content and case evidence in order to test how our findings generalized across contexts. All trial stimuli followed the general format of a civil trial. That is, participants first read a brief, neutral case summary. Then, they read opening statements from the plaintiff and defense attorneys. Next, each side presented witnesses, with the plaintiff presenting their case first. Finally, participants read closing statements from the plaintiff and defense attorneys. For each case, we relied on the verdict forms and jury instructions from the real case to develop the liability and damage award questions.

Study 1 involved a medical malpractice misdiagnosis. In that case, the plaintiff alleged that the defendant doctor negligently misdiagnosed her husband’s aortic rupture, causing his death. The defense argued that the plaintiff’s husband was in poor health and refused testing. Study 2 involved an insurance bad faith claim. The plaintiff alleged that her insurance company acted in bad faith by failing to pay her the amount owed after a car accident. The defense argued that the plaintiff’s attorney failed to cooperate with the intent of creating a bad faith claim. Study 3 involved a wrongful birth. The plaintiff alleged that the defendant, a genetic testing company, negligently failed to identify that her unborn child was at risk of cystic fibrosis. She alleged that she would have terminated the pregnancy if she had received accurate information. The defense argued that the plaintiff’s doctor had failed to order the correct test, absolving the company of blame. The average reading time across all three trial stimuli was 23 (*SD* = 11) minutes (Study 1: *M* = 19, *SD* = 9; Study 2: *M* = 22, *SD* = 10; Study 3: *M* = 27, *SD* = 12).

#### 2.2.3. Measures

The complete measures are reported in the original manuscript. The measures that we analyzed here are found at https://osf.io/685qa/ (accessed on 15 July 2025). The demographic information included gender, age, race and ethnicity, yearly income, marital status, and parental status. Then, after reading the case, participants completed a dichotomous verdict (“Liable” or “Not Liable”). Then, participants who judged that the defendant was *liable* were asked to indicate their damage awards. Participants reported their damage awards in both numbers and words. When the words and numbers differed, we found that most mistakes were due to adding or omitting a zero. In these cases, we converted the numeric values to match the written-out values.

Then, participants completed a Negative Emotions Scale (α = 0.91). The scale comprised 3 5-point Likert-type items measuring participants’ negative emotions (i.e., resentment, moral outrage, and disgust) towards the defendant.

Then, participants completed a Plaintiff Agreement Scale and a Defense Agreement Scale. Because we wanted to explore how emotions towards the defendant related to agreement with case evidence that was *unrelated* to the defendant, we limited our analyses here to items that were unrelated to the defendant’s behavior. We replicated the analyses with the full scales (Supplemental Materials pp. 14–20, Tables S10–S13, Figures S8 and S9).

The Plaintiff Agreement Scale and a Defense Agreement Scale were each based on case-specific content. In Study 1, the Plaintiff Agreement Scale comprised 3 items (α = 0.58) and included items like “Mrs. Woods [the plaintiff] lost at least 20 years with her husband”. The Defense Agreement Scale comprised 5 items (α = 0.72) and included items like “Mr. Woods’ [the plaintiff’s] aortic tear would have been discovered if he had not rejected x-rays and other tests when he was at the hospital”. In Study 2, the Plaintiff Agreement Scale comprised 2 items (α = 0.66) and included items like “The fact that the plaintiff, Ms. Bari, met with the insurance adjuster, Michelle Humphrey, shows she was being cooperative”. The Defense Agreement Scale comprised 6 items (α = 0.90) and included items like “Ms. Bari [the plaintiff] did not disclose to Allstate that she had hired an expert witness because she was setting up a bad faith claim”. In Study 3, the Plaintiff Agreement Scale comprised 4 items (α = 0.60) and included items like “If Pamela [the plaintiff] had been given the results of the Carrier test, she would have terminated the pregnancy”. The Defense Agreement Scale comprised 6 items (α = 0.59) and included items like “Robbie [the plaintiffs’ son] has fewer medical expenses than the Buddings [the plaintiffs] allege because he can cover his expenses through Obamacare”. All scale items were 6-point items assessing the extent to which participants agreed with the evidence from Strongly Disagree to Strongly Agree.

### 2.3. Data Analysis

Statistics were done using R (v4.2.3; R Core Team, 2023), the tidyverse (v2.0; Wickham et al., 2019), the emmeans (v.1.10; Lenth, 2024), the EnvStats (v2.7; Millard, 2013) and the psych (v2.3.3; Revelle, 2023) packages.

#### 2.3.1. Assumption Checks

As expected, verdict (a binary variable) was not normally distributed. Thus, we used logistic regressions when analyzing verdict.

We analyzed damage awards from participants who voted liable (*n* = 1241, 61%) and followed the same procedures for removing outliers as described in the original manuscript (for details, see Supplemental Materials p. 1). However, even after removing outliers, these data were not normally distributed, as is typical with monetary compensation (E. Greene et al., 2001) and were skewed (skewness = 0.85, kurtosis = −0.08, Shapiro-Wilk W = 0.80, *p* < .001). For this reason, we followed a similar procedure when analyzing these data as we took in the original manuscript and conducted generalized linear regressions that assumed a gamma distribution (i.e., gamma regressions).

#### 2.3.2. Preliminary Analysis Plan

We began by testing whether the persuasiveness of evidence depended on case and the party that the evidence favored. We did this by conducting a 3 (Case: Medical Malpractice Misdiagnosis, Insurance Bad Faith, Wrongful Birth) by 2 (Party: Plaintiff or Defense) Analysis of Variance (ANOVA) on agreement with case evidence). As this was a preliminary analysis, we summarize these results below and report the details of the analysis in Supplemental Materials (pp. 1–2, Table S1, Figure S1).

#### 2.3.3. Direct Effects (Hypothesis 1)

We tested hypothesis 1a using a logistic regression that included the Negative Emotions Scale and the case covariates as predictors of verdict. We tested hypothesis 1b using a gamma regression that included the Negative Emotions Scale and the case covariates as predictors of damage awards. We report the direct effect here and include the full regression tables in Supplemental Materials (p. 3, Tables S2 and S3).

#### 2.3.4. Indirect Effects (Hypotheses 2 and 3)

To test hypothesis 2a and 3a, we conducted a moderated mediation model examining the indirect effect of the Negative Emotions Scale on verdict through two simultaneous mediators: the Plaintiff Agreement Scale and the Defense Agreement Scale. This model also included case as a covariate and judicial rehabilitation as a moderator to examine whether the indirect effects of negative emotions on case judgments through plaintiff and defense evidence were diminished for those exposed to judicial rehabilitation. This model used linear regression for the a path and logistic regression for the b path. Then, we used the methods set out by Hayes (2018) to calculate indirect effects and we used nonparametric bootstrapping procedures to bootstrap 95% confidence intervals around those indirect effects. We report all hypothesized indirect effects and the regression coefficients for each path here and include the full regression table that makes up the moderated mediation models in Supplemental Materials (pp. 3–5, Table S4).

To test hypotheses 2b and 3b, we conducted a moderated mediation model examining the indirect effect of the Negative Emotions Scale on damage award through two simultaneous mediators: the Plaintiff Agreement Scale and the Defense Agreement Scale. This models also included cases as a covariate and judicial rehabilitation as a moderator to examine whether the indirect effects of negative emotions on case judgments through plaintiff and defense evidence were diminished for those exposed to judicial rehabilitation. This model used linear regression for the a path and gamma regression for the b path Again, we used the methods set out by Hayes (2018) to calculate indirect effects and nonparametric bootstrapping procedures to bootstrap 95% confidence intervals around those indirect effects. In addition to accounting for skew, these analyses produce mean ratio coefficients (reported as exp[β]), which allow us to interpret the estimates in a way that is similar to an Odds Ratio. We report all hypothesized indirect effects and the regression coefficients for each path here and include the full regression tables that make up the moderated mediation models in Supplemental Materials (pp. 3–5, Table S5).

#### 2.3.5. Supplemental Analyses

As discussed above, to reduce redundancy and increase statistical power, here, we report analyses that are collapsed across cases. We report the results of each analysis described in this section separately for each case in Supplemental Materials (pp. 5–14, Tables S6–S9, Figures S2–S7). In addition to measuring agreement with evidence that is unrelated to the defendant’s behavior, we also measured agreement with evidence that was related to the defendant’s behavior. Here, we focus on the results of the analyses focusing on evidence that is unrelated to the defendant’s behavior. We also examined whether these analyses replicated when we included evidence that was related to the defendant’s behavior in the scales. All results described are replicated below. We report the full results of these analyses in Supplemental Materials (pp. 14–20, Tables S10–S13, Figures S8 and S9).

## 3. Results

### 3.1. Preliminary Analyses

The plaintiff evidence was significantly more persuasive than the defense evidence in the Bad Faith and Wrongful Birth cases, but the defense evidence was significantly more persuasive than the plaintiff evidence in the Medical Malpractice case (see Supplemental Materials pp. 1–2, Table S1, and Figure S1 for details). This indicates that the findings discussed below generalize across diverse cases of varying strength. However, because there are overall differences in case strength, we controlled for case in all of the analyses reported below. Descriptive statistics of all variables are provided in Table 2.

### 3.2. Direct Effect Hypotheses (Hypothesis 1)

#### 3.2.1. Verdicts (Hypothesis 1a)

We first tested the theoretical direct path from emotions to verdicts via a logistic regression that included the Negative Emotions Scale and the case covariates as predictors of verdict. Consistent with our hypotheses, there was a significant effect of the Negative Emotions Scale on verdict, β = 1.25, *SE* = 0.07, 95% CIs [1.11, 1.39]. Feeling more negative emotions toward the defendant was associated with greater likelihood of a pro-plaintiff verdict.

#### 3.2.2. Damage Awards (Hypothesis 1b)

As with verdict, we tested the theoretical direct path from emotions to verdicts via a gamma regression that included the Negative Emotions Scale and the case covariates as predictors of verdict. Consistent with the results for verdict and our hypotheses, there was a significant effect of the Negative Emotions Scale on damage awards, β = 0.13, *SE* = 0.02, 95% CIs [0.10, 0.17]. Feeling more negative emotions toward the defendant was associated with awarding greater damage awards.

### 3.3. Indirect Effect Hypotheses (Hypotheses 2 and 3)

#### 3.3.1. Verdicts

Next, we tested hypotheses 2a and 3a by conducting a moderated mediation model examining the indirect effect of the Negative Emotions Scale on verdict through two simultaneous mediators: the Plaintiff Agreement Scale and the Defense Agreement Scale. These models also included cases as covariates and judicial rehabilitation as a moderator to examine whether the indirect effects of negative emotions on case judgments, cultivated through plaintiff and defense evidence, were diminished for those exposed to judicial rehabilitation.

**Figure 2:**
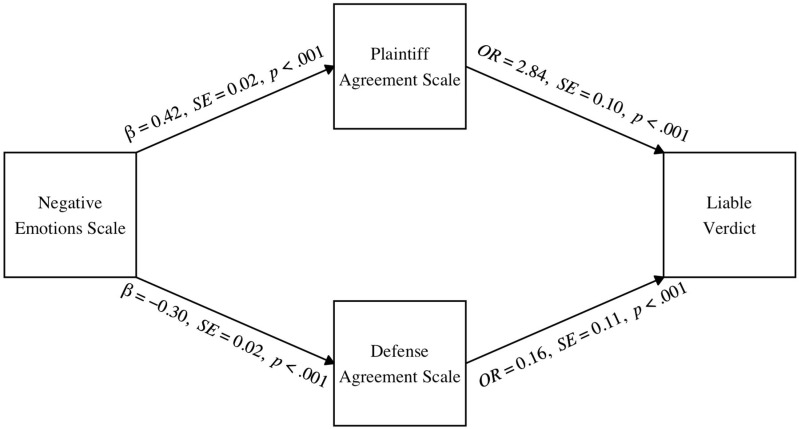
The indirect effect of negative emotions towards the defendant on verdict through agreement with plaintiff and defense evidence. *Note.* The moderated mediation model was tested using non-parametric bootstrapping methods in R. Solid lines indicate significant pathways and dotted lines indicate nonsignificant pathways.

Of importance to our theoretical predictions (Hypothesis 2a), there was a significant indirect effect of the Negative Emotions Scale on verdict through the Plaintiff Agreement Scale, β*_indirect_* = 0.42, *SE* = 0.001, 95% CIs [0.34, 0.51]. Also consistent with Hypothesis 2a, there was a significant indirect effect of the Negative Emotions Scale on verdict through the Defense Agreement Scale, β*_indirect_* = 0.53, *SE* = 0.001, 95% CIs [0.44, 0.62]. As shown in Figure 2, increased negative emotions towards the defendant predicted increased agreement with pro-plaintiff evidence that was unrelated to the defendant’s behavior and decreased agreement with pro-defense evidence that was unrelated to the defendant’s behavior. In turn, agreeing more with the plaintiff evidence and less with the defense evidence was associated with an increased likelihood of voting liable. Consistent with Hypothesis 3a, judicial rehabilitation did not moderate either indirect effect (pro-plaintiff evidence: β*_index_* = −0.05, *SE* = 0.001, 95% CIs [−0.13, 0.02]; pro-defense evidence: β*_index_* = 0.05, *SE* = 0.001, 95% CIs [−0.09, 0.019]).

#### 3.3.2. Damage Awards

**Figure 3:**
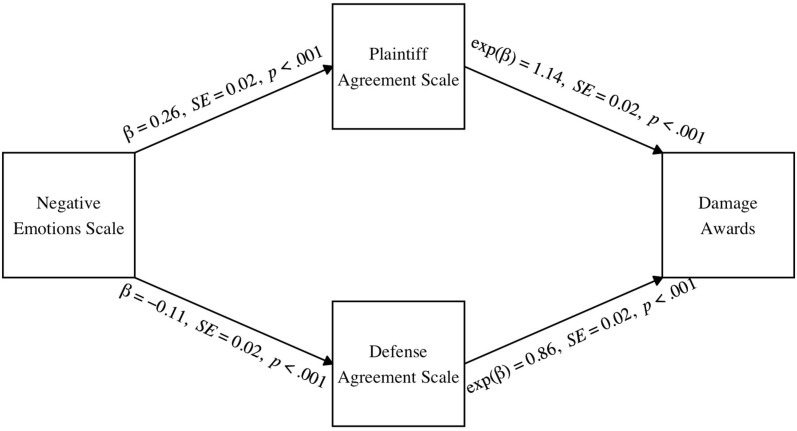
The indirect effect of negative emotions towards the defendant on damage awards through agreement with plaintiff and defense evidence. *Note.* The moderated mediation model was tested using non-parametric bootstrapping methods in R. Solid lines indicate significant pathways and dotted lines indicate nonsignificant pathways.

Of importance to our theoretical predictions (Hypothesis 2b), and consistent with the results for verdicts, there was a significant indirect effect of the Negative Emotions Scale on damages through the Plaintiff Agreement Scale, β*_indirect_* = 0.04, *SE* = 0.0001, 95% CIs [0.02, 0.05]. Also consistent with Hypothesis 2b, there was a significant indirect effect of the Negative Emotions Scale on damages through the Defense Agreement Scale, β*_indirect_* = 0.02, *SE* = 0.0001, 95% CIs [0.01, 0.03]. As shown in Figure 3, increased negative emotions towards the defendant predicted increased agreement with pro-plaintiff evidence that was unrelated to the defendant’s behaviors and decreased agreement with pro-defense evidence that was unrelated to the defendant’s behaviors. In turn, agreeing more with the plaintiff and less with the defense evidence was associated with increased damage awards. Consistent with Hypothesis 3b, judicial rehabilitation did not moderate either indirect effect (pro-plaintiff evidence: β*_index_* = −0.004, *SE* < 0.001, 95% CIs [−0.02, 0.01]; pro-defense evidence: β*_index_* = 0.02, *SE* < 0.001, 95% CIs [−0.001, 0.03]).

## 4. Discussion

In three studies, we explored the ways in which negative emotions towards the defendant relate to how they evaluated plaintiff and defense evidence—despite the fact that the evidence was not related to the defendant or his behavior. When participants reported increased negative emotions towards the defendant, they evaluated the evidence in a more anti-defendant direction. In turn, the more they evaluated the evidence in an anti-defendant direction, the more likely they were to blame the defendant and find him liable and the more money they gave the plaintiff. We found support for the Coherence Model of Cognitive Consistency in legal judgments: Jurors’ emotional responses, their review of the evidence, and their case judgments are not independent and isolated components of a decision. Instead, mock jurors’ emotions, evaluation of the evidence, and decisions are all inextricably intertwined. The more contempt, disgust and moral outrage mock jurors felt about a defendant, the more they pulled their evaluation of unrelated evidence—for example, how they judged the plaintiff’s actions—in line with that vitriol, perhaps to justify voting liable and making the defendant pay more in damages. If mock jurors were angry with the defendant, they tended to be in more agreement with the evidence that a plaintiff’s wife had lost many years with her husband or that the plaintiff would have chosen to terminate a pregnancy if she had learned that she was a Cystic Fibrosis carrier. And that agreement matters: Agreement with that evidence predicted more liable verdicts and greater damage awards. Thus, it is not as simple as that if jurors are angry at the defendant they will judge behaviors more harshly—they will also judge *other* evidence in a more pro-plaintiff/anti-defendant light, such as judging the plaintiff’s suffering and behavior more favorably.

This is very different from traditional characterizations of jurors’ emotions directly driving their decisions in lazy or heuristic ways that bypasses or replaces evidence evaluation entirely—or Darrow’s argument that jury decisions are driven by their feelings towards a defendant, rather than the facts. Indirect effects could be more insidious and persistent because they can occur when jurors are trying to be diligent about reviewing all the evidence. Our data suggest that their emotional reactions and the diligent review of evidence are intertwined. Perhaps because emotional responses contribute to a selective focusing (perhaps unconsciously) on pro-plaintiff evidence and a selective discounting of defense evidence—all the while, believing that they were working hard to be impartial and review the evidence thoroughly. In support, the effect was not decreased when jurors were exposed to judicial rehabilitation that called for them to be aware of potential bias and judging the case in a fair and impartial way.

Our findings generalized across different case scenarios and evidence strength. We even found that negative emotions about the defendant predicted decreased agreement with defense evidence in the case where defense evidence was more persuasive than plaintiff evidence, overall.

### 4.1. Theoretical Contributions

The current research makes theoretical contributions by informing the indirect relationship between emotions and judgments. This research provides support for an indirect effect of emotions on decision-making, consistent with models of emotion and legal decision making (Feigenson & Park, 2006; Salerno, 2021) and blame more generally (Culpable Control Model, Alicke, 2000; Coherence-Based Reasoning, Simon & Read, 2025). Mock jurors who report more negative emotions towards the defendant evaluate the evidence in a pro-plaintiff manner. This research also adds to literature on the coherence effect. Prior research suggests that hot cognitions, such as emotion, can influence both blame attributions (e.g., Goldberg et al., 1999; Rudolph et al., 2004) and the ways in which people interpret information (e.g., Kahan, 2013). This research suggests that emotion, evidence evaluation, and judgments are all linked to each other. When participants feel increased negative emotions towards the defendant, their evaluation of the unrelated evidence might shift in a way that is consistent with their negative emotions (i.e., favoring the plaintiff) and their case judgments, in turn, might favor the plaintiff. However, we should note that this research does not test the causal relationship between negative emotions, case judgments, and verdicts. It is possible that case judgments contribute to and increase feelings of negative emotions. Indeed, this is consistent with prior literature on the coherence effect, which suggests that the relationship between emotions and cognitive appraisal is bidirectional (Simon & Read, 2025). Instead, we provide support for the coherence effect such that participants’ emotions, evidence evaluations, and case judgments are interrelated and align to form a coherent, consistent picture.

This research also contributes to psycho-legal research by increasing our understanding of the relationship between emotions and evidence evaluation. For example, the relationship between emotionally evocative pretrial publicity and decisions (Kramer et al., 1990; Phalen et al., 2021) might be mediated by how jurors interpret the facts. Similarly, emotions might influence how jurors respond to conflicting damage award recommendations from both sides or how jurors evaluate conflicting experts. That is, it is possible that when jurors are angry at the defendant, they might view the plaintiff’s damage award recommendation as more reasonable or evaluate the defense’s expert as less credible. Importantly, some psycho-legal manipulations (e.g., injury severity) might also inadvertently increase mock jurors’ emotions, which might, in turn, influence those findings. In sum, these findings have broad implications for a number of effects observed in jury decision-making work as jurors’ emotions might influence how jurors interpret a variety of scenarios and facts. Future research should consider how the manipulation inadvertently influences emotions and test how emotions moderate well-established findings in jury decision-making.

### 4.2. Legal Implications

Consistent with previous research (e.g., Salerno & Phalen, 2019; Simon et al., 2015), these findings demonstrate that, contrary to popular belief in the legal system, jurors’ emotions are not a substitute for a logical evaluation of the evidence but rather relate to the way that jurors evaluate the evidence. Participants’ negative emotional responses to the defendant are not only associated with increased pro-plaintiff judgments, as prior research suggests, but also with a pro-plaintiff evaluation of the evidence unrelated to the defendant’s actions, like whether a child with Cystic Fibrosis would face serious medical complications or if a plaintiff’s attorney was setting up a bad faith claim. This finding might be especially important in cases that involve punitive damages. For example, if a plaintiff presents evidence that supports a claim for punitive damages (i.e., that the defendant acted with malice) and makes jurors angry toward the defendant, those emotions might also relate to how jurors evaluate evidence that is unrelated to the claim for punitive damages, like assessments of the plaintiff’s injury severity.

In contrast to previous research on damage awards (e.g., Feigenson et al., 2001; Fishfader et al., 1996), we found a significant relationship between emotions and damage awards, through mock jurors’ interpretation of plaintiff and defense evidence. There are several potential explanations for this effect. First, our sample size was much larger than the sample size in the previous studies; the prior studies that found no relationship between emotions and damage awards both used fewer than 200 participants. Given the wide variation in damage awards and the relatively small effect sizes that we found here, it’s possible that the previous studies were not powered to detect an effect of emotions on damages. Further, we investigated the relationship between emotions and damage awards both directly and indirectly, through mock jurors’ interpretation of the evidence. In this way, we qualify Fishfader and colleagues’ (1996) suggestion that damage awards are driven by the facts, by demonstrating that the interpretation of those facts are also predicted by emotions towards a defendant.

Importantly, this research suggests that participating in judicial rehabilitation does not diminish the relationship between emotions and evidence evaluation and case judgments. Although the manipulation was not originally designed for this purpose, it enabled us to test and show that making mock jurors more focused on their potential biases and motivated to review the evidence in an impartial way did not diminish the relationship between their emotional response to the defendant and how they evaluated other evidence and decided the case. This finding is particularly concerning, given the legal system’s assumption that judicial rehabilitation will serve to detect and excuse potential jurors who are unable to regulate their own biases. These findings join other research (e.g., Crocker & Kovera, 2010; Schuller et al., 2015) in suggesting that judicial rehabilitation is not an effective tool for ensuring jurors are unbiased. Additionally, this finding challenges the assumption that judges can instruct jurors to set their biases aside, such as letting their emotions bias their evaluation of evidence. Even if jurors are told to be unbiased and are acting diligently to ensure that their emotions are not driving their decisions, those same emotions might be, unconsciously changing the way that jurors evaluate the evidence. Of course, a manipulation designed to specifically mention emotion-based biases might have been more effective.

This research has important implications for attorneys in that they ought to consider how the emotional valence of a case might relate to jurors’ interpretation of the facts. On the one hand, if an attorney knows that the opposing side has the more emotionally compelling case, they might choose to recommend a settlement. On the other hand, if an attorney knows that their case is emotionally compelling, it might serve them to rely on those emotions. This research also has important implications for judges who might be searching for a way to mitigate emotion-driven bias. It might not be enough to simply instruct jurors to remain unbiased or to decide a case without sympathy or prejudice. Instead, jury instructions should consider the nuanced ways in which emotions relate to both judgments and evidence evaluation. Given that emotions and evidence evaluation are related, future research might focus on testing interventions that reduce the strength of this relationship.

### 4.3. Strengths, Limitations, and Future Directions

We examined the relationships between negative emotions towards a defendant, plaintiff and defense evidence, and verdicts across three legal cases, with a wide variety of facts, strengths, and weaknesses. The indirect effect of negative moral emotions toward the defendant on case judgments existed regardless of which side’s case was stronger, across cases with high and low damage awards, and across three very different sets of facts and defendants.

The stimuli were designed to mimic a real civil trial as closely as possible, given that the study was online. Participants read extensive trial information including opening and closing statements from both plaintiff and defense lawyers as well as a presentation of evidence including direct- and cross-examination of witnesses. Additionally, the three cases varied widely in their emotional content. Specifically, the case in Study 2 (the Insurance Bad Faith Case) was less emotionally charged than the other two cases but we found similar results. However, there are limitations to the case materials that we presented. While we used three very different cases, three cases cannot represent the full range of possible civil lawsuits. Future research should examine the effects of emotions in other civil contexts, particularly in contexts were there are allegations of actual bad acts (and therefore increased negative emotions towards the defendant), rather than simple negligence. Further, because we intended for the cases to closely reflect the information that jurors would receive in a real trial, we did not follow the design rules recommended for moral dilemma tasks. Although we argue that this design increases the external validity of the work, it does make it more difficult to draw comparisons to research on moral dilemmas. Future research should investigate how other dimensions of moral dilemmas’ research, such as framing or expression style, influence jury decision-making.

Additionally, while a review of jury simulations suggests that experimental juror research can inform and replicate real juror judgments (Bornstein et al., 2017), we recognize the differences between this research and real civil trials. On the one hand, real trials, with live testimony from sympathetic victims, might be even more emotionally charged than these case summaries, which might exacerbate the effects that we found here. On the other hand, real trials take place over a number of days, which might blunt the emotional effect of any one piece of evidence. Furthermore, deliberation might alter the relationship between emotions and evidence evaluations. That is, deliberation might reduce the effect of emotions on the evaluation of evidence because jurors have the ability to “vent” their emotions during that deliberation. Alternatively, deliberation might exacerbate emotions through emotion contagion (Hatfield et al., 1993) or group polarization (Sunstein, 2002). Future research should investigate the effect of deliberation on the relationship between jurors’ emotions and the way that they evaluate the evidence.

Further, this research is limited in that we only measured negative emotions toward the defendant, rather than general affect. Our intention was for this work to act as a first step examining how feeling emotions directly related to one of the parties relates to changes in evidence evaluation and case judgments. However, in doing so, we do not measure or test how general affect might relate to evidence evaluation, nor do we test how these effects differ across different valences and levels of arousal. Future research should expand this work to test different emotions and affect more generally.

Future research should also consider the role of culture and personal beliefs in the relationship between emotions and evidence evaluation. Indeed, some research suggests that feeling moral outrage exacerbates the effects of personal beliefs on case judgments (e.g., Peter-Hagene & Bottoms, 2017; Peter-Hagene & Ratliff, 2021). This might be because emotional jurors are selectively favoring case facts that align with their personal beliefs. Similarly, research suggests that emotions are influenced both by cultural background and context (Pugh et al., 2022). Cultural background and the cultural norms relating to emotion might be an important moderator in the theoretical model we have proposed. Future research should examine how these relationships vary based on culture.

This research is also limited in that we are unable to draw causal conclusions about the relationships investigated here. We did not experimentally manipulate participants’ emotions and therefore, we can only conclude that negative emotions towards a defendant are correlated with a pro-plaintiff evaluation of the evidence. It is possible that agreeing more with the plaintiff’s evidence causes increased negative emotions towards a defendant or that there is a cyclical relationship between negative emotions towards the defendant and a pro-plaintiff evaluation of the evidence. That is, consistent with Coherence Model of Cognitive Consistency, it is possible that the relationship between emotions and evidence evaluation is bidirectional and cannot be easily confined to a unidirectional causal model. Instead, it might be that negative emotions towards the defendant causes a pro-plaintiff evaluation of the evidence and that pro-plaintiff evaluation of the evidence, in turn, confirms and increases negative feelings towards the defendant and that this cycle repeats until a juror has reached a high-enough level of certainty to commit to a verdict. In this paper, we do not aim to draw causal conclusions about our findings. Rather, we aim to contribute to the development of a theoretical causal model and serve as a potential first step for future causal research (Rohrer et al., 2022).

Additionally, because we found that participants’ emotions about the defendant predict their evaluation of evidence that was unrelated to the defendants’ actions, this research still has important legal and theoretical contributions. That is, even without a causal test, the fact that participants’ emotions about the defendant are related to their evaluation of unrelated evidence adds to research on the coherence effect and provides important information to lawyers about how jurors might incorporate their emotions into how they evaluate other, unrelated evidence and, ultimately, their case judgments. Rather than providing a test of a causal relationship, this research acts as a proof of concept examining the potential relationships between emotions and evaluations of evidence. Future research should test these relationships experimentally and with computer simulations to draw more concrete causal conclusions. Future research should also attempt to investigate the potential cyclical relationship between emotions and the evaluation of case evidence more directly.

Despite these limitations, this research provides important preliminary evidence for the relationship between negative emotions towards the defendant, the evaluation of evidence, and ultimate case judgments. While future research should investigate the potential causal relationship between emotions and the evaluation of the evidence in civil cases, we hope that this important first step inspires future research to think about coherence-based models of juror decision making.

## 5. Conclusions

Legal actors have long been concerned with the impact of jurors’ perceptions of the defendant on case judgments. However, this research suggests that there is an important indirect relationship between emotions towards the defendant and judgments that cannot be ignored. Specifically, across three cases, this research demonstrates that feeling more negative emotions towards the defendant predicts an increase in agreement with plaintiff evidence and a decrease in agreement with defense evidence, even when the evidence is unrelated to the defendant or the defendant’s behavior. This pro-plaintiff evaluation of the case evidence is associated with an increase in the likelihood that mock jurors vote liable and give higher damage awards. In other words, Clarence Darrow did not have it quite right in 1935 when he said the facts of a case are relatively unimportant. Instead, mock jurors’ emotional responses towards a defendant relate to their interpretation of the facts and, in turn, their decisions—while maintaining the impression that their decision was impartial and based on facts. Attorneys should think more carefully about the interrelated nature of jurors’ emotional reactions to a defendant, jurors’ evaluation of the evidence, and jurors’ case judgments.

## Figures and Tables

**Table 1:** Sample demographics.

Demographic		*n* or Mean	% or Standard Deviation
Age		39.87	12.61
Gender			
	Men	775	37.97
	Women	1266	62.03
Race/Ethnicity			
	White (Non-Hispanic)	1580	77.41
	Black/African American	181	8.87
	Native American	6	0.29
	Asian	123	6.03
	Hispanic	119	5.83
	Other	32	1.57
Education			
	Less than high school	9	0.44
	High school degree or equivalent	178	8.72
	Some college but no degree	479	23.47
	Technical or Vocational school	62	3.04
	Associate degree	251	12.30
	Bachelor degree	769	37.68
	Graduate degree	260	12.74
	Doctorate degree	33	1.62
Parental Status			
	Parent	1083	53.06
	Not a Parent	958	46.94
Marital Status			
	Married	826	40.47
	Never Married	802	39.29
	Divorced	212	10.39
	Widowed	72	3.53
	Partnered	87	4.26
	Separated	42	2.06
Income		59,377.63	43,181.41

**Table 2:** Descriptive statistics of all measures.

Measure	All Cases *N* = 2041	Medical Malpractice Misdiagnosis (Study 1, *n* = 713)	Insurance Bad Faith (Study 2, *n* = 677)	Wrongful Birth (Study 3, *n* = 651)
Negative Emotions Scale	2.27 (1.21)	1.98 (1.17)	2.26 (1.11)	2.60 (1.26)
Plaintiff Agreement Scale	4.08 (1.05)	3.75 (1.00)	4.70 (0.92)	3.80 (0.93)
Defense Agreement Scale	3.74 (1.03)	4.40 (0.80)	3.30 (1.12)	3.47 (0.75)
Liable Verdict	1246 (61%)	268 (38%)	481 (71%)	497 (76%)
Damage Award (Median)	5,000,000.00 (500,000.00, 30,000,000.00)	15,000,000.00 (5,000,000.00, 25,000,000.00)	450,000.00 (300,000.00, 500,000.00)	30,000,000.00 (10,500,000.00, 37,000,000.00)
Damage Award (Mean)	13,896,213.75 (15,824,630.69)	16,514,074.54 (11,787,776.63)	427,818.30 (164,166.72)	25,540,445.07 (15,408,670.16)

*Note.* The verdict indicates the number and percentage of liable verdicts in each condition. For each continuous measure, the means are reported with the standard deviation in parentheses. For damage awards, both mean and median are reported. Medians are reported with the first and third quartiles in parentheses. Shading added for readability.

## Data Availability

The original data presented in the study are openly available on osf at https://osf.io/685qa/ (accessed 15 July 2025).

## Supplementary Materials

The following supporting information can be downloaded at: https://www.mdpi.com/article/10.3390/bs15070965/s1, Table S1: Effect of Case and Party on Agreement with the Evidence; Figure S1: Effect of Case and Party on Agreement with the Evidence; Table S2: Regression Table for the Direct Effect of Negative Emotions on Verdict; Table S3: Regression Table for the Direct Effect of Negative Emotions on Damage Awards; Table S4: Regression Table for the Indirect Effect of Negative Emotions on Verdict; Table S5: Regression Table for the Indirect Effect of Negative Emotions on Damage Awards; Table S6: Indirect Effects of Negative Emotions Towards the Defendant on Verdict Through Agreement with Plaintiff and Defense Evidence Broken Down by Study; Table S7: Regression Table for the Indirect Effect of Negative Emotions on Verdicts Broken Down by Study; Figure S2: Study 1: Indirect Effects of Negative Emotions towards the Defendant on Verdict through Agreement with Plaintiff and Defense Evidence.; Figure S3: Study 2: Indirect Effects of Negative Emotions towards the Defendant on Verdict through Agreement with Plaintiff and Defense Evidence; Figure S4: Study 3: Indirect Effects of Negative Emotions towards the Defendant on Verdict through Agreement with Plaintiff and Defense Evidence; Table S8: Indirect Effects of Negative Emotions towards the Defendant on Damage Awards through Agreement with Plaintiff and Defense Evidence Broken Down by Study; Table S9: Regression Table for the Indirect Effect of Negative Emotions on Damage Awards Broken Down by Study; Figure S5: Study 1: Indirect Effects of Negative Emotions towards the Defendant on Damage Awards through Agreement with Plaintiff and Defense Evidence; Figure S6: Study 2: Indirect Effects of Negative Emotions towards the Defendant on Damage Awards through Agreement with Plaintiff and Defense Evidence; Figure S7: Study 3: Indirect Effects of Negative Emotions towards the Defendant on Damage Awards through Agreement with Plaintiff and Defense Evidence; Table S10: Indirect Effects of Negative Emotions towards the Defendant on Verdicts through Agreement with both Related and Unrelated Plaintiff and Defense Evidence; Table S11: Regression Table for the Indirect Effect of Negative Emotions on Verdict Through All Inferences; Figure S8: Indirect Effects of Negative Emotions towards the Defendant on Verdicts through Agreement with both Related and Unrelated Plaintiff and Defense Evidence; Table S12: Indirect Effects of Negative Emotions towards the Defendant on Damage Awards through Agreement with both Related and Unrelated Plaintiff and Defense Evidence; Table S13: Regression Table for the Indirect Effect of Negative Emotions on Damage Awards Through all Inference; Figure S9: Indirect Effects of Negative Emotions towards the Defendant on Damage Awards through Agreement with both Related and Unrelated Plaintiff and Defense Evidence.
