# Supplementary material for: Jurymen Seldom Rule Against a Person That They Like: The Relationship Between Emotions Towards a Defendant, the Understanding of Case Facts, and Juror Judgments in Civil Trials
Source: Behav Sci (Basel). 2025 Jul 16;15(7):965. doi: 10.3390/bs15070965 (PMC12292101; doi:10.3390/bs15070965)
Supplement: Supplementary file 1 [file behavsci-15-00965-s001.zip › behavsci-3696627-supplementary.pdf]

Table of Contents

Preliminary Analyses 1

Preliminary Analyses 1

Table S1 *Effect of Case and Party on Agreement with the Evidence* 2

Figure S1 *Effect of Case and Party on Agreement with the Evidence* 2

Full Regression Tables 3

Direct Effects 3

Table S2 *Regression Table for the Direct Effect of Negative Emotions on Verdict* 3

Table S3 *Regression Table for the Direct Effect of Negative Emotions on Damage Awards* 3

Indirect Effects 3

Table S4 *Regression Table for the Indirect Effect of Negative Emotions on Verdict* 4

Table S5 *Regression Table for the Indirect Effect of Negative Emotions on Damage Awards* 5

Results Broken Down by Individual Studies 5

Verdicts 7

Table S6 *Indirect Effects of Negative Emotions towards the Defendant on Verdict through Agreement with Plaintiff and Defense Evidence Broken Down by Study.* 7

Table S7 *Regression Table for the Indirect Effect of Negative Emotions on Verdicts Broken Down by Study* 8

Figure S2 *Study 1: Indirect Effects of Negative Emotions towards the Defendant on Verdict through Agreement with Plaintiff and Defense Evidence.* 9

Figure S3 *Study 2: Indirect Effects of Negative Emotions towards the Defendant on Verdict through Agreement with Plaintiff and Defense Evidence.* 9

Figure S4 *Study 3: Indirect Effects of Negative Emotions towards the Defendant on Verdict through Agreement with Plaintiff and Defense Evidence.* 10

Damages 11

Table S8 *Indirect Effects of Negative Emotions towards the Defendant on Damage Awards through Agreement with Plaintiff and Defense Evidence Broken Down by Study.* 11

Table S9 *Regression Table for the Indirect Effect of Negative Emotions on Damage Awards Broken Down by Study* 12

Figure S5 *Study 1: Indirect Effects of Negative Emotions towards the Defendant on Damage Awards through Agreement with Plaintiff and Defense Evidence.* 13

Figure S6 *Study 2: Indirect Effects of Negative Emotions towards the Defendant on Damage Awards through Agreement with Plaintiff and Defense Evidence.* 13

Figure S7 *Study 3: Indirect Effects of Negative Emotions towards the Defendant on Damage Awards through Agreement with Plaintiff and Defense Evidence.* 14

Moderated Mediation Analyses with Evidence that is both Related and Unrelated to the Defendant 14

Verdicts 15

Table S10 *Indirect Effects of Negative Emotions towards the Defendant on Verdicts through Agreement with both Related and Unrelated Plaintiff and Defense Evidence.* 15

Table S11 *Regression Table for the Indirect Effect of Negative Emotions on Verdict Through All Inferences* 16

Figure S8. *Indirect Effects of Negative Emotions towards the Defendant on Verdicts through Agreement with both Related and Unrelated Plaintiff and Defense Evidence.* 17

Damages 18

Table S12 *Indirect Effects of Negative Emotions towards the Defendant on Damage Awards through Agreement with both Related and Unrelated Plaintiff and Defense Evidence.* 18

Table S13 *Regression Table for the Indirect Effect of Negative Emotions on Damage Awards Through all Inferences* 19

Figure S9. *Indirect Effects of Negative Emotions towards the Defendant on Damage Awards through Agreement with both Related and Unrelated Plaintiff and Defense Evidence.* 20

Preliminary Analyses

Outlier Analysis

As described on lines 401–403 in the main manuscript, we began by visually inspecting box plots for all three studies combined and each individual case. Then, we conducted Rosner's tests (1983) on each of the case-specific data files to confirm that the values were outliers. The Rosner's tests identified 7 outliers across the three cases (Study 1 $n = 3$; Study 2 $n = 2$; Study 3 $n = 2$). Using winsorization (Tukey, 1962), we replaced these outliers with the next highest non-excluded value from their specific case (Study 1: 51,370,000; Study 2: 1,000,000; Study 3: 80,000,000).

Preliminary Analyses

As discussed on lines 409–415 and 461–468 of the main manuscript, we ran a 3 (Case: Medical Malpractice Misdiagnosis, Insurance Bad Faith, Wrongful Birth) by 2 (Party: Plaintiff or Defense) Analysis of Variance (ANOVA) on agreement with case evidence (Table S1, Figure S1). Broadly, we found that persuasiveness does depend on case. This indicates that the findings generalize across diverse cases of varying strength.

Table S1

Effect of Case and Party on Agreement with the Evidence

Parameter	*F*(dfn, dfd)	*p*-value	*Effect Size*	95% *CI*
Overall Sample				
Main Effect of Case	**86.27 (2, 4074)**	**< .001**	**0.04**	**0.03, 0.05**
Main Effect of Party	**138.86 (1, 4074)**	**< .001**	**0.03**	**0.02, 0.04**
Interaction	**421.82 (2, 4074)**	**< .001**	**0.17**	**0.12, 0.19**
Simple Effects				
Study 1: Plaintiff versus Defense Inferences	**767.57 (1, 4074)**	**< .001**	**–1.51**	**–1.62, –1.39**
Study 2: Plaintiff versus Defense Inferences	**1753.97 (1, 4074)**	**< .001**	**0.70**	**0.59, 0.80**
Study 3: Plaintiff versus Defense Inferences	**40.97 (1, 4074)**	**< .001**	**–0.35**	**–0.46, –0.25**

Note. Confidence intervals that do not cross one and statistically significant differences at $p < .05$ are bolded.

Figure S1

Effect of Case and Party on Agreement with the Evidence

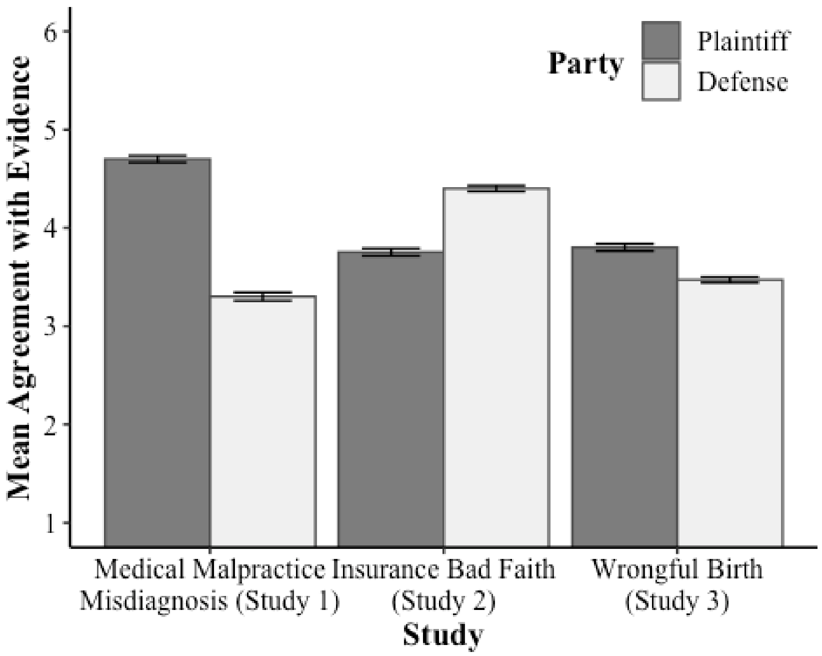

Note. Error bars represent ± 1 standard error.

Full Regression Tables

Direct Effects

In the main text, we report the direct effects of negative emotions on verdict and damage awards on lines 474–488. Here, we report the full regression tables for verdict (Table S2) and for damage awards (Table S3).

Table S2

Regression Table for the Direct Effect of Negative Emotions on Verdict

Parameter	β	*SE*	*p*-value	*Odds Ratio*	95% *CI*
**Intercept**	**0.69**	**.06**	**< .001**	**2.00**	**1.78, 2.24**
**Emotion**	**1.25**	**.07**	**< .001**	**3.48**	**3.03, 3.99**
**Case [Medical Malpractice]**	**–0.69**	**.06**	**< .001**	**0.50**	**0.45, 0.57**
Case [Wrongful Birth]	0.03	.06	.62	1.03	0.91, 1.17

Note. Confidence intervals that do not cross one and statistically significant differences at *p* < .05 are bolded. Case is coded with the Insurance Bad Faith case as the reference group.

Table S3

Regression Table for the Direct Effect of Negative Emotions on Damage Awards

Parameter	β	*SE*	*p*-value	*exp*(β)	95% *CI*
**Intercept**	**15.37**	**.02**	**< .001**	**4,732,523.22**	**4,589,949.83, 4,879,525.22**
**Emotion**	**0.13**	**.02**	**< .001**	**1.14**	**1.11, 1.18**
**Case [Medical Malpractice]**	**1.47**	**.02**	**< .001**	**4.34**	**4.19, 4.49**
**Case [Wrongful Birth]**	**1.98**	**.02**	**< .001**	**7.26**	**7.02, 7.52**

Note. Confidence intervals that do not cross one and statistically significant differences at *p* < .05 are bolded. Case is coded with the Insurance Bad Faith case as the reference group.

Indirect Effects

In the main text, we report the indirect effects of negative emotions on verdict and damage awards through two simultaneous mediators: agreement with the plaintiff and agreement

with the defense on lines 489–533. Here, we report the full regression tables for verdict (Table S4) and for damage awards (

Parameter	β	*SE*	*p*-value	*Odds Ratio*	95% *CI*
			Agreement with the Plaintiff		
**Intercept**	**4.08**	**.02**	**< .001**	**—**	**4.05, 4.12**
**Emotion**	**0.42**	**.02**	**< .001**	**—**	**0.38, 0.46**
Judicial Rehabilitation	–0.02	.02	.25		–0.06, 0.02
**Case [Medical Malpractice]**	**–0.45**	**.02**	**< .001**	**—**	**–0.45, –0.36**
**Case [Wrongful Birth]**	**–0.47**	**.02**	**< .001**	**—**	**–0.52, –0.43**
Emotion X Judicial Rehabilitation	–0.03	.02	.18		–0.06, 0.01
			Agreement with the Defense		
**Intercept**	**3.74**	**.02**	**< .001**	**—**	**3.70, 3.78**
**Emotion**	**–0.30**	**.02**	**< .001**	**—**	**–0.34, –0.26**
Judicial Rehabilitation	0.02	.02	.29		–0.02 0.06
**Case [Medical Malpractice]**	**0.49**	**.02**	**< .001**	**—**	**0.45, 0.53**
**Case [Wrongful Birth]**	**0.12**	**.02**	**< .001**	**—**	**0.07, 0.16**
Emotion X Judicial Rehabilitation	–0.01	.02	.48		–0.05, 0.02
			Verdict		
**Intercept**	**1.05**	**.08**	**< .001**	**2.87**	**2.45, 3.36**
**Emotion**	**0.84**	**.09**	**< .001**	**2.31**	**1.95, 2.75**
**Agreement with the Plaintiff**	**1.04**	**.10**	**< .001**	**2.84**	**2.34, 3.44**
**Agreement with the Defense**	**–1.81**	**.11**	**< .001**	**0.16**	**0.13, 0.20**
**Case [Medical Malpractice]**	**0.20**	**.09**	**.02**	**1.22**	**1.03, 1.46**
**Case [Wrongful Birth]**	**0.55**	**.09**	**< .001**	**1.74**	**1.44, 2.09**

Note. Confidence intervals that do not cross one and statistically significant differences at $p < .05$ are bolded. Case is coded with the Insurance Bad Faith case as the reference group.

Table S5).

Table S4

Regression Table for the Indirect Effect of Negative Emotions on Verdict

Parameter	β	*SE*	*p*-value	*Odds Ratio*	95% *CI*
			Agreement with the Plaintiff		
**Intercept**	**4.08**	**.02**	**< .001**	—	**4.05, 4.12**
**Emotion**	**0.42**	**.02**	**< .001**	—	**0.38, 0.46**
Judicial Rehabilitation	–0.02	.02	.25		–0.06, 0.02
**Case [Medical Malpractice]**	**–0.45**	**.02**	**< .001**	—	**–0.45, –0.36**
**Case [Wrongful Birth]**	**–0.47**	**.02**	**< .001**	—	**–0.52, –0.43**
Emotion X Judicial Rehabilitation	–0.03	.02	.18		–0.06, 0.01
			Agreement with the Defense		
**Intercept**	**3.74**	**.02**	**< .001**	—	**3.70, 3.78**
**Emotion**	**–0.30**	**.02**	**< .001**	—	**–0.34, –0.26**
Judicial Rehabilitation	0.02	.02	.29		–0.02 0.06
**Case [Medical Malpractice]**	**0.49**	**.02**	**< .001**	—	**0.45, 0.53**
**Case [Wrongful Birth]**	**0.12**	**.02**	**< .001**	—	**0.07, 0.16**
Emotion X Judicial Rehabilitation	–0.01	.02	.48		–0.05, 0.02
			Verdict		
**Intercept**	**1.05**	**.08**	**< .001**	**2.87**	**2.45, 3.36**
**Emotion**	**0.84**	**.09**	**< .001**	**2.31**	**1.95, 2.75**
**Agreement with the Plaintiff**	**1.04**	**.10**	**< .001**	**2.84**	**2.34, 3.44**
**Agreement with the Defense**	**–1.81**	**.11**	**< .001**	**0.16**	**0.13, 0.20**
**Case [Medical Malpractice]**	**0.20**	**.09**	**.02**	**1.22**	**1.03, 1.46**
**Case [Wrongful Birth]**	**0.55**	**.09**	**< .001**	**1.74**	**1.44, 2.09**

Note. Confidence intervals that do not cross one and statistically significant differences at $p < .05$ are bolded. Case is coded with the Insurance Bad Faith case as the reference group.

Table S5

Regression Table for the Indirect Effect of Negative Emotions on Damage Awards

Parameter	β	*SE*	*p*-value	*exp*(β)	95% *CI*
			Agreement with the Plaintiff		
**Intercept**	**4.51**	**.02**	**< .001**	**—**	**4.46, 4.55**
**Emotion**	**0.26**	**.02**	**< .001**	**—**	**0.21, 0.30**
Judicial Rehabilitation	–0.04	.02	.09		–0.08, 0.22
**Case [Medical Malpractice]**	**–0.27**	**.02**	**< .001**	**—**	**–0.31, –0.22**
**Case [Wrongful Birth]**	**–0.50**	**.02**	**< .001**	**—**	**–0.55, –0.45**
Emotion X Judicial Rehabilitation	–0.01	.02	.73		–0.05, 0.04
			Agreement with the Defense		
**Intercept**	**3.21**	**.02**	**< .001**	**—**	**3.17, 3.25**
**Emotion**	**–0.11**	**.02**	**< .001**	**—**	**–0.15, –0.07**
Judicial Rehabilitation	0.02	.02	.41		–0.02, 0.06
**Case [Medical Malpractice]**	**0.45**	**.02**	**< .001**	**—**	**0.40, 0.49**
**Case [Wrongful Birth]**	**0.26**	**.02**	**< .001**	**—**	**0.21, 0.30**
Emotion X Judicial Rehabilitation	–0.05	.02	.05		–0.09, 0.01
			Damage Awards		
**Intercept**	**15.35**	**.02**	**< .001**	**4,647,324.83**	**4,507,263.83 4,791,738.15**
**Emotion**	**0.09**	**.02**	**< .001**	**1.09**	**1.06, 1.13**
**Agreement with the Plaintiff**	**0.13**	**.02**	**< .001**	**1.14**	**1.10, 1.18**
**Agreement with the Defense**	**–0.16**	**.02**	**< .001**	**0.86**	**0.83, 0.89**
**Case [Medical Malpractice]**	**1.58**	**.02**	**< .001**	**4.83**	**4.65, 5.02**
**Case [Wrongful Birth]**	**2.09**	**.02**	**< .001**	**8.06**	**7.75, 8.39**

Note. Confidence intervals that do not cross one and statistically significant differences at $p < .05$ are bolded. Case is coded with the Insurance Bad Faith case as the reference group.

Results Broken Down by Individual Studies

As described on lines 450–453 of the main paper, in order to increase power and avoid redundancy, we reported the indirect effect results collapsed across studies in the main paper. We

report the results broken down by study here. We conducted moderated mediation analyses in each study examining the indirect effects of negative emotions towards the defendant on verdicts (Table S6–Table S7; Figures Figure S2;Figure S3Figure S4) and damage awards (Table S8Table S9, Figures Figure S5Figure S6Figure S7) through agreement with unrelated plaintiff and defense evidence for both mock jurors who were exposed to judicial rehabilitation and those who were not. Judicial rehabilitation did not moderate any of the indirect effects (all confidence intervals cross zero). With three exceptions (the indirect effect of the Negative Emotions Scale on Damage Awards through the Defense Agreement Scale in the Medical Malpractice Misdiagnosis case) and main effects of judicial rehabilitation on agreement with the plaintiff in the Insurance Bad Faith Case), the pattern of all results was the same as when we collapse across cases.

Verdicts

Table S6

Indirect Effects of Negative Emotions towards the Defendant on Verdict through Agreement with Plaintiff and Defense Evidence Broken Down by Study.

Effect	Medical Malpractice Misdiagnosis (Study 1)			Insurance Bad Faith (Study 2)			Wrongful Birth (Study 3)		
	Beta	SE	95% CI	Beta	SE	95% CI	Beta	SE	95% CI
Total Effect	**1.72**	**.13**	**1.48, 1.98**	**0.81**	**0.11**	**0.60, 1.03**	**1.04**	**0.13**	**0.80, 1.30**
Mediator: Plaintiff Agreement Scale	**0.42**	**.001**	**0.29, 0.58**	**0.22**	**0.001**	**0.12, 0.34**	**0.39**	**0.001**	**0.28, 0.52**
Mediator: Defense Agreement Scale	**0.42**	**.001**	**0.31, 0.56**	**0.69**	**0.002**	**0.49, 0.94**	**0.23**	**0.001**	**0.15, 0.33**
Index of Moderated Mediation: Plaintiff Agreement Scale	–0.05	.001	–0.16, 0.06	–0.02	.001	–0.12, 0.08	–0.09	.001	–0.22, 0.03
Index of Moderated Mediation: Defense Agreement Scale	0.05	.001	–0.09, 0.19	0.09	.003	–0.27, 0.46	0.01	.001	–0.13, 0.14

Note. Significant indirect effects are **bolded**.

Table S7

Regression Table for the Indirect Effect of Negative Emotions on Verdicts Broken Down by Study

	Medical Malpractice Misdiagnosis (Study 1)					Insurance Bad Faith (Study 2)					Wrongful Birth (Study 3)				
Parameter	β	*SE*	*p*-value	*Odds Ratio*	95% *CI*	β	*SE*	*p*-value	*Odds Ratio*	95% *CI*	β	*SE*	*p*-value	*Odds Ratio*	95% *CI*
Agreement with the Plaintiff															
Intercept	**3.75**	**0.03**	**< .001**	**—**	**3.69, 3.82**	**4.70**	**.03**	**< .001**	**—**	**4.63, 4.77**	**3.80**	**.03**	**< .001**	**—**	**3.73, 3.86**
Emotion	**0.53**	**0.03**	**< .001**	**—**	**0.46, 0.59**	**0.28**	**.03**	**< .001**	**—**	**0.22, 0.35**	**0.41**	**.03**	**< .001**	**—**	**0.34, 0.47**
Judicial Rehabilitation	0.01	0.03	.86	—	−0.06, 0.07	**−0.11**	**.03**	**.002**	**—**	**−0.17, −0.04**	0.03	.03	.38	—	−0.04, 0.09
Emotion X Judicial Rehabilitation	−0.03	0.03	.35	—	−0.09, 0.03	−0.010	.03	.77	—	−0.08, 0.06	−0.05	.03	.16	—	−0.11, 0.02
Agreement with the Defense															
Intercept	**4.40**	**0.03**	**< .001**	**—**	**4.35, 4.45**	**3.30**	**.04**	**< .001**	**—**	**3.22, 3.38**	**3.47**	**.03**	**< .001**	**—**	**3.42, 3.53**
Emotion	**−0.36**	**0.03**	**< .001**	**—**	**−0.41, −0.30**	**−0.32**	**.04**	**< .001**	**—**	**−0.40, −0.24**	**−0.20**	**.03**	**< .001**	**—**	**−0.26, −0.15**
Judicial Rehabilitation	−0.01	0.03	.72	—	−0.06, 0.04	0.08	.04	.06	—	−0.005, 0.16	−0.004	.03	.89	—	−0.06, 0.05
Emotion X Judicial Rehabilitation	−0.02	0.03	.42	—	−0.08, 0.03	−0.02	.04	.61	—	−0.10, 0.06	−0.005	.03	.86	—	−0.06, 0.05
Verdict															
Intercept	**−0.70**	**0.12**	**< .001**	**0.50**	**0.39, 0.62**	**2.08**	**.19**	**< .001**	**8.01**	**5.51, 11.65**	**1.97**	**.16**	**< .001**	**7.18**	**5.26, 9.80**
Emotion	**1.27**	**0.14**	**< .001**	**3.56**	**2.69, 4.72**	**0.48**	**.17**	**.004**	**1.62**	**1.16, 2.25**	**0.55**	**.15**	**< .001**	**1.74**	**1.30, 2.33**
Agreement with the Plaintiff	**0.93**	**0.15**	**< .001**	**2.54**	**1.89, 3.42**	**0.76**	**.17**	**< .001**	**2.15**	**1.53, 3.02**	**1.14**	**.16**	**< .001**	**3.14**	**2.31, 4.28**
Agreement with the Defense	**−1.12**	**0.15**	**< .001**	**0.33**	**0.24, 0.44**	**−2.65**	**.24**	**< .001**	**0.07**	**0.04, 0.11**	**−1.06**	**.14**	**< .001**	**0.35**	**0.26, 0.46**

Note. Confidence intervals that do not cross one and statistically significant differences at $p < .05$ are bolded

Figure S2

Study 1: Indirect Effects of Negative Emotions towards the Defendant on Verdict through Agreement with Plaintiff and Defense Evidence.

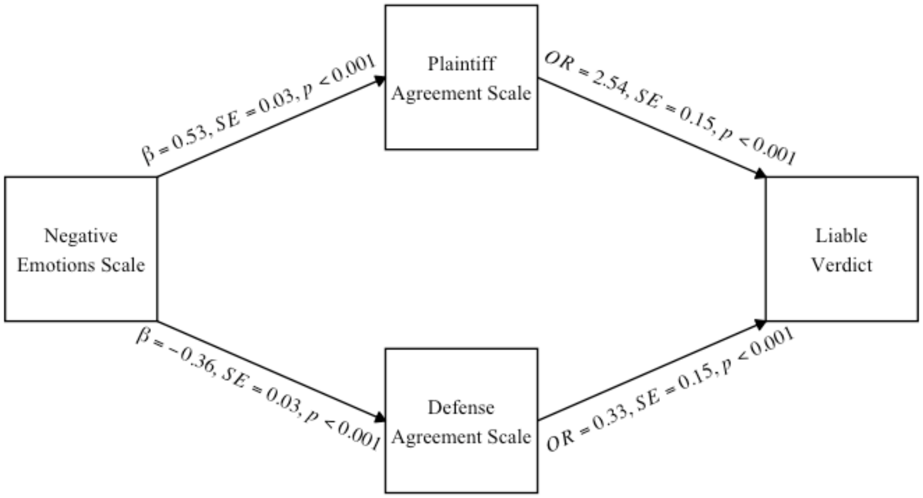

Note. The moderated mediation model was tested using non-parametric bootstrapping methods in R. Solid lines indicate significant pathways and dotted lines indicate nonsignificant pathways.

Figure S3

Study 2: Indirect Effects of Negative Emotions towards the Defendant on Verdict through Agreement with Plaintiff and Defense Evidence.

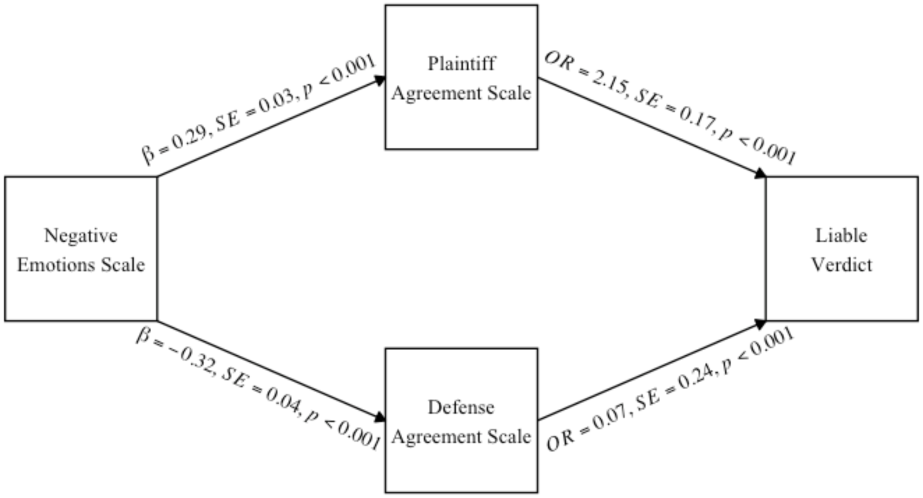

Note. The moderated mediation model was tested using non-parametric bootstrapping methods in R. Solid lines indicate significant pathways and dotted lines indicate nonsignificant pathways.

Figure S4

Study 3: Indirect Effects of Negative Emotions towards the Defendant on Verdict through Agreement with Plaintiff and Defense Evidence.

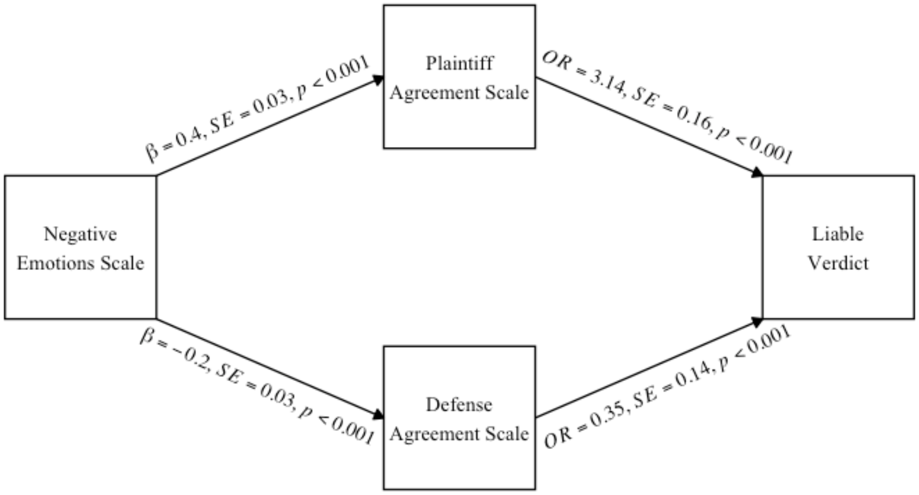

Note. The moderated mediation model was tested using non-parametric bootstrapping methods in R. Solid lines indicate significant pathways and dotted lines indicate nonsignificant pathways.

Damages

Table S8

Indirect Effects of Negative Emotions towards the Defendant on Damage Awards through Agreement with Plaintiff and Defense Evidence Broken Down by Study.

Effect	Medical Malpractice Misdiagnosis (Study 1)			Insurance Bad Faith (Study 2)			Wrongful Birth (Study 3)		
	Beta	SE	95% CI	Beta	SE	95% CI	Beta	SE	95% CI
Total Effect	**0.23**	**0.04**	**0.15, 0.31**	**0.09**	**0.02**	**0.06, 0.13**	**0.12**	**0.03**	**0.07, 0.17**
Mediator: Plaintiff Agreement Scale	**0.09**	**0.0003**	**0.05, 0.14**	**0.01**	**0.0001**	**0.003, 0.02**	**0.03**	**0.0001**	**0.01, 0.05**
Mediator: Defense Agreement Scale	0.01	0.0001	–0.0002, 0.03	**0.01**	**0.0001**	**0.002, 0.02**	**0.03**	**0.0002**	**0.01, 0.05**
Index of Moderated Mediation: Plaintiff Agreement Scale	−0.01	<.001	−0.06, 0.03	<–.001	<.001	−0.01, 0.009	−0.004	<.001	−0.02, 0.01
Index of Moderated Mediation: Defense Agreement Scale	0.01	<.001	−0.005, 0.04	0.009	<.001	−0.004, 0.03	0.02	<.001	−0.02, 0.06

Note. Significant indirect effects are **bolded**.

Table S9

Regression Table for the Indirect Effect of Negative Emotions on Damage Awards Broken Down by Study

	Medical Malpractice Misdiagnosis (Study 1)					Insurance Bad Faith (Study 2)					Wrongful Birth (Study 3)				
Parameter	β	*SE*	*p*-value	*Odds Ratio*	95% *CI*	β	*SE*	*p*-value	*Odds Ratio*	95% *CI*	β	*SE*	*p*-value	*Odds Ratio*	95% *CI*
Agreement with the Plaintiff															
Intercept	**4.45**	**.05**	**< .001**	**—**	**4.35, 4.55**	**5.00**	**.03**	**< .001**	**—**	**4.94, 5.07**	**4.05**	**.04**	**< .001**	**—**	**3.98, 4.12**
Emotion	**0.39**	**.05**	**< .001**	**—**	**0.29, 0.49**	**0.15**	**.03**	**< .001**	**—**	**0.07, 0.21**	**0.28**	**.04**	**< .001**	**—**	**0.21, 0.35**
Judicial Rehabilitation	−0.06	.05	.28	—	−0.16, 0.05	**−0.09**	**.03**	**.009**	**—**	**−0.16, −0.02**	0.02	.04	.64	—	−0.05, 0.09
Emotion X Judicial Rehabilitation	−0.03	.05	.61	—	−0.13, 0.08	−0.002	.03	.96	—	−0.07, 0.07	−0.02	.04	.59	—	−0.09, 0.05
Agreement with the Defense															
Intercept	**3.84**	**.05**	**< .001**	**—**	**3.75, 3.93**	**2.79**	**.04**	**< .001**	**—**	**2.72, 2.86**	**3.28**	**.03**	**< .001**	**—**	**3.22, 3.34**
Emotion	**−0.14**	**.05**	**.004**	**—**	**−0.23, −0.05**	**−0.11**	**.04**	**.002**	**—**	**−0.18, −0.04**	**−0.10**	**.03**	**.001**	**—**	**−0.16, −0.04**
Judicial Rehabilitation	0.001	.05	.98	—	−0.09, 0.09	0.05	.04	.15	—	−0.02, 0.12	−0.007	.03	.81	—	−0.07, 0.05
Emotion X Judicial Rehabilitation	−0.08	.05	.08	—	−0.17, 0.01	−0.05	.04	.15	—	−0.12, 0.02	−0.03	.03	.40	—	−0.09, 0.03
Damage Awards															
Intercept	**16.56**	**.04**	**< .001**	**15615284.66**	**14369010.79, 16969652.17**	**12.96**	**.02**	**< .001**	**423255.51**	**409711.69, 437247.04**	**17.01**	**.03**	**< .001**	**24477270.22**	**23124008.50, 25909727.43**
Emotion	**0.13**	**.05**	**.007**	**1.14**	**1.04, 1.25**	**0.07**	**.02**	**< .001**	**1.08**	**1.04, 1.11**	**0.07**	**.03**	**.02**	**1.07**	**1.01, 1.14**
Agreement with the Plaintiff	**0.25**	**.05**	**< .001**	**1.28**	**1.16, 1.41**	**0.06**	**.02**	**.002**	**1.06**	**1.02, 1.10**	**0.11**	**.03**	**< .001**	**1.11**	**1.04, 1.18**
Agreement with the Defense	−0.09	.04	.06	0.92	**0.84, 1.00**	**−0.08**	**.02**	**< .001**	**0.92**	**0.89, 0.96**	**−0.25**	**.03**	**< .001**	**0.78**	**0.73, 0.82**

Note. Confidence intervals that do not cross one and statistically significant differences at $p < .05$ are bolded

Figure S5

Study 1: Indirect Effects of Negative Emotions towards the Defendant on Damage Awards through Agreement with Plaintiff and Defense Evidence.

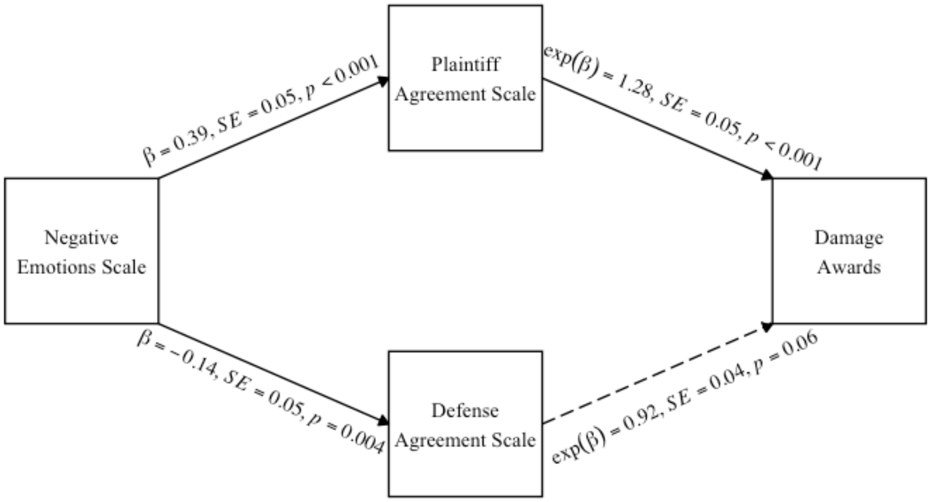

Note. The moderated mediation model was tested using non-parametric bootstrapping methods in R. Solid lines indicate significant pathways and dotted lines indicate nonsignificant pathways.

Figure S6

Study 2: Indirect Effects of Negative Emotions towards the Defendant on Damage Awards through Agreement with Plaintiff and Defense Evidence.

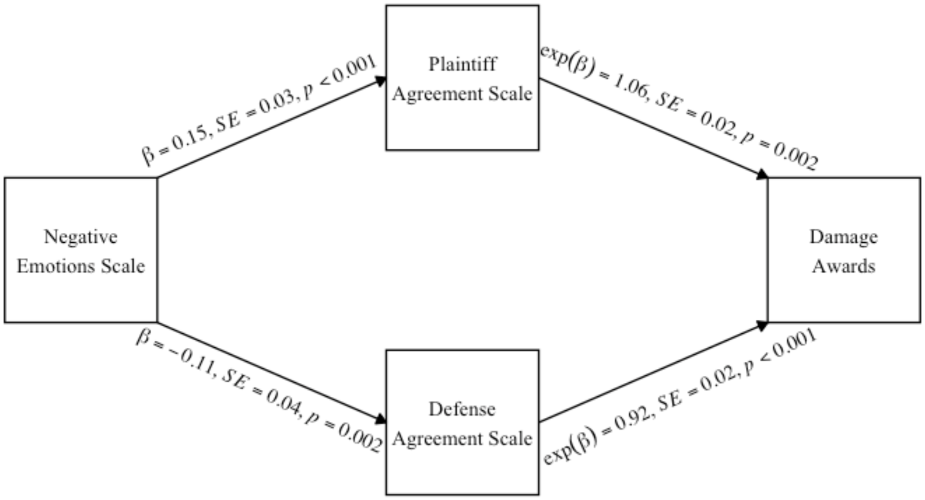

Note. The moderated mediation model was tested using non-parametric bootstrapping methods in R. Solid lines indicate significant pathways and dotted lines indicate nonsignificant pathways.

Figure S7

Study 3: Indirect Effects of Negative Emotions towards the Defendant on Damage Awards through Agreement with Plaintiff and Defense Evidence.

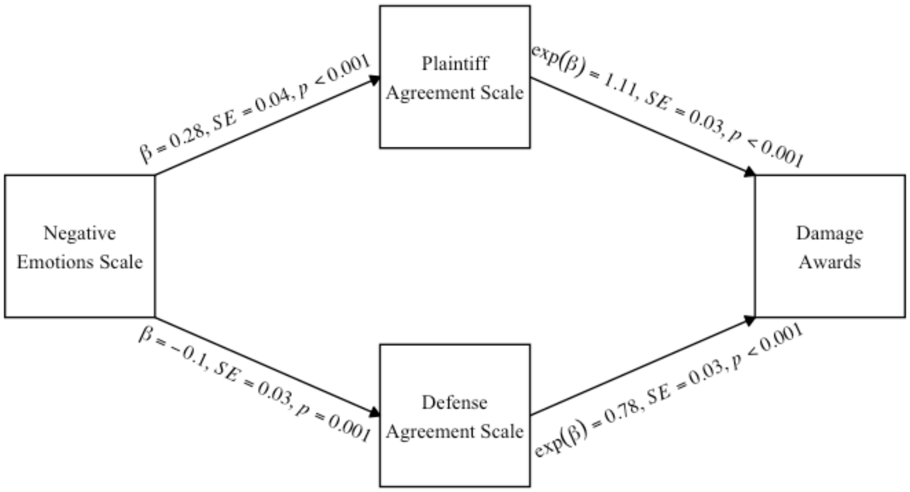

Note. The moderated mediation model was tested using non-parametric bootstrapping methods in R. Solid lines indicate significant pathways and dotted lines indicate nonsignificant pathways.

Moderated Mediation Analyses with Evidence that is both Related and Unrelated to the Defendant

In the main manuscript, we report moderated mediation analyses focusing on evidence that is unrelated to the defendant. However, as described on lines 453–459 of the main paper, we also measured agreement with evidence that was related to the defendant's behaviors. We report the results of moderated mediation analyses examining the indirect effects of negative emotions towards the defendant on verdicts (Table S10Table S11, Figure S8) and damage awards (Table S12Table S13, Figure S9) through agreement with related and unrelated plaintiff and defense evidence for both mock jurors who were exposed to judicial rehabilitation and those who were not. Judicial rehabilitation did not moderate any of the indirect effects (all confidence intervals cross zero). The pattern of all results was the same as the analyses reported in the main manuscript.

Verdicts

Table S10

Indirect Effects of Negative Emotions towards the Defendant on Verdicts through Agreement with both Related and Unrelated Plaintiff and Defense Evidence.

Effect	β	*SE*	95% *CI*
Total Effect	**1.25**	**0.07**	**1.11, 1.39**
Mediator: Plaintiff Agreement Scale	**0.92**	**0.001**	**0.78, 1.08**
Mediator: Defense Agreement Scale	**0.78**	**0.001**	**0.65, 0.92**
Index of Moderated Mediation: Plaintiff Agreement Scale	−0.11	.001	−0.24, 0.02
Index of Moderated Mediation: Defense Agreement Scale	−0.03	.001	−0.18, 0.13

Note. Significant indirect effects are **bolded**.

Table S11

Regression Table for the Indirect Effect of Negative Emotions on Verdict Through All Inferences

Parameter	β	*SE*	*p*-value	*Odds Ratio*	95% *CI*
Agreement with the Plaintiff					
**Intercept**	4.17	.02	< .001	—	4.14, 4.21
**Emotion**	0.45	.02	< .001	—	0.42, 0.48
Judicial Rehabilitation	0.007	.02	.66	—	−0.03, 0.04
**Case [Medical Malpractice]**	−0.39	.02	< .001	—	−0.43, −0.35
**Case [Wrongful Birth]**	−0.19	.02	< .001	—	−0.22, −0.15
Emotion X Judicial Rehabilitation	−0.03	.02	.12	—	−0.06, 0.007
Agreement with the Defense					
**Intercept**	3.61	.02	< .001	—	3.57, 3.64
**Emotion**	−0.36	.02	< .001	—	−0.40, −0.33
Judicial Rehabilitation	0.03	.02	.15	—	−0.009, 0.06
**Case [Medical Malpractice]**	0.38	.02	< .001	—	0.34, 0.42
**Case [Wrongful Birth]**	0.09	.02	< .001	—	0.05, 0.13
Emotion X Judicial Rehabilitation	0.006	.02	.75	—	−0.03, 0.04
Verdict					
**Intercept**	1.29	.10	< .001	3.64	3.00, 4.41
**Emotion**	0.48	.11	< .001	1.62	1.31, 1.99
**Agreement with the Plaintiff**	1.94	.15	< .001	6.94	5.21, 9.26
**Agreement with the Defense**	−2.08	.15	< .001	0.12	0.09, 0.17
**Case [Medical Malpractice]**	0.32	.11	.003	1.37	1.12, 1.69
**Case [Wrongful Birth]**	0.42	.10	< .001	1.52	1.24, 1.86

Note. Confidence intervals that do not cross one and statistically significant differences at $p < .05$ are bolded. Case is coded with the Insurance Bad Faith case as the reference group.

Figure S8.

Indirect Effects of Negative Emotions towards the Defendant on Verdicts through Agreement with both Related and Unrelated Plaintiff and Defense Evidence.

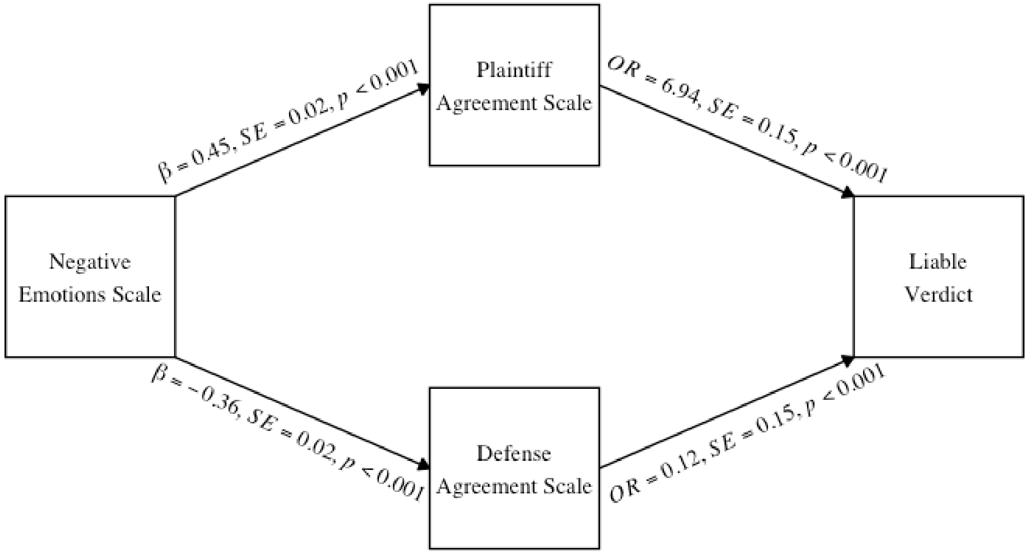

Note. The moderated mediation model was tested using non-parametric bootstrapping methods in R. Solid lines indicate significant pathways and dotted lines indicate nonsignificant pathways.

Damages

Table S12

Indirect Effects of Negative Emotions towards the Defendant on Damage Awards through Agreement with both Related and Unrelated Plaintiff and Defense Evidence.

Effect	β	*SE*	95% *CI*
Total Effect	**0.13**	**0.02**	**0.10, 0.17**
Mediator: Plaintiff Agreement Scale	**0.04**	**0.0001**	**0.02, 0.06**
Mediator: Defense Agreement Scale	**0.03**	**0.0001**	**0.02, 0.04**
Index of Moderated Mediation: Plaintiff Agreement Scale	−0.006	< .001	−0.02, 0.005
Index of Moderated Mediation: Defense Agreement Scale	0.007	< .001	−0.009, 0.02

Note. Significant indirect effects are **bolded**.

Table S13

Regression Table for the Indirect Effect of Negative Emotions on Damage Awards Through all Inferences

Parameter	β	*SE*	*p*-value	*exp*(β)	95% *CI*
			Agreement with the Plaintiff		
Intercept	**4.72**	**.02**	**< .001**	—	**4.69, 4.76**
Emotion	**0.23**	**.02**	**< .001**	—	**0.19, 0.26**
Judicial Rehabilitation	−0.007	.02	.68	—	−0.04, 0.03
Case [Medical Malpractice]	**−0.27**	**.02**	**< .001**	—	**−0.31, −0.24**
Case [Wrongful Birth]	**−0.22**	**.02**	**< .001**	—	**−0.26, −0.19**
Emotion X Judicial Rehabilitation	−0.02	.02	.32	—	−0.05, 0.02
			Agreement with the Defense		
Intercept	**3.04**	**.02**	**< .001**	—	**3.01, 3.08**
Emotion	**−0.14**	**.02**	**< .001**	—	**−0.18, −0.10**
Judicial Rehabilitation	0.03	.02	.13	—	−0.009, 0.06
Case [Medical Malpractice]	**0.31**	**.02**	**< .001**	—	**0.27, 0.35**
Case [Wrongful Birth]	**0.19**	**.02**	**< .001**	—	**0.15, 0.23**
Emotion X Judicial Rehabilitation	−0.02	.02	.35	—	−0.05, 0.02
			Damage Awards		
Intercept	**15.35**	**.02**	**< .001**	**4644725.16**	**4505280.30, 4788486.04**
Emotion	**0.07**	**.02**	**< .001**	**1.08**	**1.04, 1.11**
Agreement with the Plaintiff	**0.12**	**.02**	**< .001**	**1.12**	**1.08, 1.17**
Agreement with the Defense	**−0.15**	**.02**	**< .001**	**0.86**	**0.83, 0.89**
Case [Medical Malpractice]	**1.57**	**.02**	**< .001**	**4.80**	**4.62, 4.99**
Case [Wrongful Birth]	**2.05**	**.02**	**< .001**	**7.77**	**7.49, 8.06**

Note. Confidence intervals that do not cross one and statistically significant differences at $p < .05$ are bolded. Case is coded with the Insurance Bad Faith case as the reference group.

Figure S9.

Indirect Effects of Negative Emotions towards the Defendant on Damage Awards through Agreement with both Related and Unrelated Plaintiff and Defense Evidence.

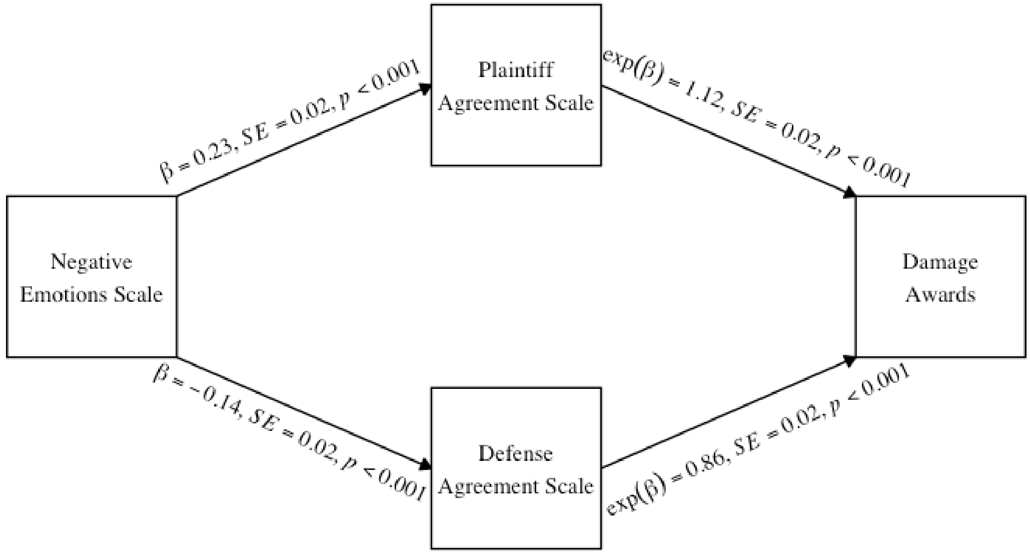

Note. The moderated mediation model was tested using non-parametric bootstrapping methods in R. Solid lines indicate significant pathways and dotted lines indicate nonsignificant pathways.
